# Dynamic Changes in the Microbiome of Rice During Shoot and Root Growth Derived From Seeds

**DOI:** 10.3389/fmicb.2020.559728

**Published:** 2020-09-08

**Authors:** Mengying Wang, Alexander W. Eyre, Michael R. Thon, Yeonyee Oh, Ralph A. Dean

**Affiliations:** ^1^Fungal Genomics Laboratory, Department of Entomology and Plant Pathology, Center for Integrated Fungal Research, North Carolina State University, Raleigh, NC, United States; ^2^Spanish-Portuguese Institute for Agricultural Research (CIALE), University of Salamanca, Villamayor, Spain

**Keywords:** rice, seed and seedling, microbiome, diversity, driving factors

## Abstract

Microbes form close associations with host plants including rice as both surface (epiphytes) and internal (endophytes) inhabitants. Yet despite rice being one of the most important cereal crops agriculturally and economically, knowledge of its microbiome, particularly core inhabitants and any functional properties bestowed is limited. In this study, the microbiome in rice seedlings derived directly from seeds was identified, characterized and compared to the microbiome of the seed. Rice seeds were sourced from two different locations in Arkansas, USA of two different rice genotypes (Katy, M202) from two different harvest years (2013, 2014). Seeds were planted in sterile media and bacterial as well as fungal communities were identified through 16S and ITS sequencing, respectively, for four seedling compartments (root surface, root endosphere, shoot surface, shoot endosphere). Overall, 966 bacterial and 280 fungal ASVs were found in seedlings. Greater abundance and diversity were detected for the microbiome associated with roots compared to shoots and with more epiphytes than endophytes. The seedling compartments were the driving factor for microbial community composition rather than other factors such as rice genotype, location and harvest year. Comparison with datasets from seeds revealed that 91 (out of 296) bacterial and 11 (out of 341) fungal ASVs were shared with seedlings with the majority being retained within root tissues. Core bacterial and fungal microbiome shared across seedling samples were identified. Core bacteria genera identified in this study such as *Rhizobium*, *Pantoea*, *Sphingomonas*, and *Paenibacillus* have been reported as plant growth promoting bacteria while core fungi such as Pleosporales, *Alternaria* and *Occultifur* have potential as biocontrol agents.

## Introduction

Macro-organisms such as plants form close interactions with microbes, which together can be considered as meta-organisms or holobionts (Berg et al., 2014). Fungi, bacteria, viruses, archaea and protista that are closely associated with plants are often referred to as the “second genome” (Berendsen et al., 2012). Different plant compartments such as roots, leaves, stems, flowers, fruits as well as seeds can all be colonized, potentially with different microbes (Berg et al., 2014). Microbes accumulate not only on the outer surfaces of plants as epiphytes but also inside plant tissues as endophytes (Turner et al., 2013). With the advent of new sequencing technologies over the past few years, the composition and possible function of these microbes, which collectively form the microbiome, associated with plants has drawn much interest (Müller et al., 2016).

Attention to microbes associated with plants has risen because they can establish beneficial, neutral or detrimental interactions of varying intimacy with their host plants (Berg et al., 2014). Beneficial microbes may promote plant growth, suppress biotic as well as abiotic stress and improve product quality. For example, various rhizobia and mycorrhizal fungi have been demonstrated to improve the acquisition of nutrients by plants (Hawkins et al., 2000; Zehr et al., 2003; Richardson et al., 2009; Miransari, 2011). Fungal endophytes such as *Neotyphodium lolii* can influence host plant CO_2_ fixation (Spiering et al., 2006). Bacteria including *Bacillus* and *Paenibacillus* are able to promote plant growth in desert agroecosystems, whereas fungi such as *Lewia* sp. can be used for rhizoremediation of hydrocarbons (Köberl et al., 2011; Cruz-Hernández et al., 2013). Unlike pathogenic microbes that cause disease on plants, microbes from *Proteobacteria, Firmicutes*, and *Actinobacteria* are known to suppress plant disease (Mendes et al., 2011).

Rice (*Oryza sativa*) is the most important cereal crop agriculturally and economically feeding over half of the world’s population. In addition, because of its relatively small genome size and molecular tractability, it has been established as a model plant for both basic and applied research (Izawa and Shimamoto, 1996; Shimamoto and Kyozuka, 2002; Rensink and Buell, 2004; Kawahara et al., 2013). Current strategies used to increase rice yield include breeding and application of chemical fertilizers and pesticides, which can be time consuming, expensive and environmental unfriendly (Khush, 2000; Peng et al., 2006; Zhang, 2007; Mano and Morisaki, 2008; Huang et al., 2017). Other environmentally conscious alternatives are in high demand such as the identification and application of beneficial microbes. Though limited research has been done, knowledge of the microbiome associated with rice is beginning to accumulate. For example, three different root niches [rhizosphere, rhizoplane (the root surface) and root endosphere] of rice were shown to carry different microbial communities including eubacteria and methanogenic archaea (Edwards et al., 2015). Rapid and selective acquisition of root-associated microbes from the soil was demonstrated (Edwards et al., 2015). In addition, *Methylobacterium* in rice shoots, *Azospirillum* and *Herbaspirillum* in rice stems and roots, and *Burkholderia* and *Rhizobium* in roots were detected (Mano and Morisaki, 2008). Similar bacteria were also found to be associated with other plants facilitating nitrogen fixation, and stress tolerance such as high osmotic pressure, dryness and gamma-ray radiation (Mano et al., 2006; Mano and Morisaki, 2008). It was also found that microbes from *Alphaproteobacteria*, *Actinobacteria*, *Pantoea*, *Exiguobacterium*, and *Bacillus* were common in the rice phyllosphere. Such microbes may have significant effects on global carbon, nitrogen and other nutrient cycles at the ecosystem level (Venkatachalam et al., 2016).

Given the abundant evidence that various microbes influence plant growth and development, considerable research focuses on understanding the microbial community to benefit modern agriculture. However, many factors influence the plant microbiome. Different agricultural practices such as tillage, drainage, intercropping, rotation, grazing and application of pesticides, fungicides as well as fertilizer can affect microbial diversity dramatically (Peiffer et al., 2013; Kato et al., 2015; Rothenberg et al., 2016; Vukicevich et al., 2016; Jenkins et al., 2017). Soil type, environmental conditions and host genotype also play important roles in shaping the microbiome assemblage. For rice, metagenomic, transcriptomic, proteomic as well as amplicon sequencing approaches used to characterize the microbial community of plants grown in soil have shown that numerous factors including environmental factors, plant age and genotype all greatly influence it’s microbiome (Knief et al., 2012; Sessitsch et al., 2012; Edwards et al., 2015). Productivity and health of agricultural systems depend greatly on the functional processes carried out by the plant-associated microbial community (Buyer et al., 1999; Hacquard, 2016).

However, it is conceivable that plants maintain a core microbiome independent from soil type, environment, host genotype, agricultural management and other factors. The concept of a core microbiome was first proposed for the human microbiome and has been further expanded to plant-associated microbes (Engelbrektson et al., 2012; Shade and Handelsman, 2012). These core microorganisms constitute a conserved subset of microbes that likely play important roles for host plants as well as for the surrounding microbial communities (Engelbrektson et al., 2012; Huse et al., 2012).

Moreover, there are also limited studies regarding microbiome variation along different life stages of plants. Reproduction is an important stage, and seeds usually contain a high diversity of microbes that can be transmitted vertically across generations (Bragina et al., 2013; Hodgson et al., 2014; Truyens et al., 2015; Shahzad et al., 2018). Seed germination is a complex process, during which the initially dormant seeds undergo physiological state changes (Ofek et al., 2011). Investigation of the microbiome temporal shift from seed to seedling as well as spatial shift from root to shoot and from tissue surface to interior may help to shed light on the interactions between the host and the associated microbiome.

The primary objective of this project was to identify the microbiome associated with rice shoots and roots and compare them with the microbiome associated with rice seeds. Furthermore, we wanted to illustrate the effect of rice tissue compartment, genotype, growth location and harvest year in shaping the microbial community. Finally, the core microbiome related with rice seedlings was also expected to be revealed. To achieve these goals, we characterized the microbial biodiversity of rice seedlings, both in shoot and root tissue, derived from seeds germinated in axenic conditions. Microorganisms associated with different rice seedling compartments (surface and endosphere of shoots and roots) were characterized by amplicon sequence of 16S for bacteria and ITS for fungi. Rice seeds from different geographic cultivation areas of different rice genotype in different harvest years were used in this study (Supplementary Table 1). The composition and population structure in seedling and root compartments were compared to those of previously published data for the seeds and seed compartments (Eyre et al., 2019). Finally, core bacterial and fungal taxa were identified.

## Materials and Methods

### Rice Seeds

Rice seeds were obtained from Dr. Yulin Jia, USDA Dale Bumpers National Rice Research Center, Stuttgart, Arkansas. Six different *japonica* rice seeds representing two rice varieties (M202 and Katy) were collected from two locations: research fields at the Dale Bumpers (DB) Research Center and the University of Arkansas (UA) in 2 years (2013 and 2014) (see Eyre et al., 2019). Seeds were enclosed in envelopes (50 g for each type of seeds) and sent through standard mail. They were stored dry at 4°C after received.

### Rice Seedling Growth

Sand (100 mL) and distilled water (40 mL) were poured into each square plant culture vessels (SPL Life Science, Incu Tissue) and autoclaved. After cooling, rice seeds were embedded into the sand and vessels sealed with 3M medi-pore tape. Each vessel contained 5–6 rice seeds and for each rice type 4 replicates were grown. Vessels were placed in an incubator at 26/20°C under a 14 h light/10 h dark cycle for 3 weeks during which time rice seeds germinated and grew to 3–4 leaf seedlings (Ding et al., 2012).

### Seedling Compartments Sample Collection

For rice seedlings, shoots and roots were separated and put into sterile 50 mL falcon centrifuge tubes using sterile tweezers and scissors. Each falcon tube contained 3–6 shoots or roots from the germinated rice seeds (root samples were first manually shaken before placing into falcon tubes in order to remove the loosely associated sand). Then 20 mL of sterile distilled water was added. The tubes were vortexed for 2 min to remove any adhering microbes, and the liquid was collected. Tubes were vortexed two more times, followed by three 1-min sonication with sterile water using a sonication probe (Microson Ultrasonic Cell Disruptor model XL2000, Misonix Incorporated New York, United States, output 7 watts) to remove tightly adhering microbes. Liquid extracts were pooled together based on different seed types to form the shoot and root surface compartment samples. The remaining shoot and root tissue were washed two more times by sonication and then placed separately in sterile tubes.

After preliminary confirmation and evaluation for bacteria and fungi existing in the four seedling compartments by plate culturing, samples for genomic DNA extraction were then processed. To extract DNA from shoot and root surface fractions (all replicates were used for DNA extraction and combined), the liquid extracts were centrifuged at 12,000 rpm for 15 min and the supernatant was removed from the pellets. Respective pellets represented the shoot surface and root surface compartments. Pellets were collected and stored at −20°C until DNA extraction (Bulgarelli et al., 2012; Engelbrektson et al., 2012; Bulgarelli et al., 2015). For shoot and root endosphere DNA samples, the remaining shoot tissue and root tissue after washing by sonication (above) were stored at −20°C until DNA extraction (Engelbrektson et al., 2012; Bulgarelli et al., 2013).

### DNA Extraction

Whole genomic DNA was extracted from the 24 different samples. The pellet collected from “shoot surface,” “root surface” samples as well as the shoot and root tissue were placed separately in sterile mortar and pestles. Liquid nitrogen was added. Samples were thoroughly ground and DNA was extracted using the “Wizard Genomic DNA Purification Kit” by Promega (Madison, WI, United States) following the provided instructions (Fadrosh et al., 2014). DNA quality and concentration were checked using the NanoDrop spectrophotometer (model ND-1000, Thermo Fisher Scientific, Waltham, MA, United States).

### 16S V3-V4 and ITS1 PCR Amplification

The amplification was carried out using primers modified from Fadrosh et al. (2014). For bacteria, a region of approximately 460 bp encompassing the V3 and V4 hypervariable regions of the 16S rRNA gene was targeted (IlluminaF: 5′-CCTACGGGNGGCWGCAG-3′ and IlluminaR: 5′-GACTACHVGGGTATCTAATCC-3′) (Klindworth et al., 2013)^1^. For fungi, the primers were used to amplify 291 ± 58 bp ITS1 region (ITS1F: 5′-CTTGGTCATTTAGAGGAAGTAA-3′ and ITS2R: 5′-GCTGCGTTCTTCATCGATGC-3′) (White et al., 1990; Gardes and Bruns, 1993; Usyk et al., 2017). Overhang adapters were added to primers for compatibility with the Nextera Index Kit (Illumina, San Diego, CA, United States).

Two stages of PCR were then conducted as described in Eyre et al. (2019). Specific index pairs were assigned to each sample following the manufacturer’s user manual. Bacterial 16S amplicon and fungal ITS amplicon coming from same sample shared the identical barcode for Mi-Seq sequencing. All 48 amplicon products (24 for bacteria and 24 for fungi) were quantified using a Bioanalyzer (Agilent 2200 TapeStation, CA, United States). Amplicons were diluted and pooled together at equimolar concentrations to ensure equal proportions of the bacterial and fungal amplifications. The prepared samples were submitted to the Genomic Sciences Laboratory at North Carolina State University for “Illumina MiSeq 300 bp Paired-End Sequencing” (Illumina, San Diego, CA, United States).

### Sequencing Data Analysis

Sequencing data obtained from the Illumina MiSeq runs was demultiplexed at the sequencing center for the 24 different samples (Supplementary Table 1) based on the barcode sequences attached to each sample. FastQC v0.11.8^2^ was then used to visualize the quality of raw sequences. Reads for each sample were further separated as bacterial and fungal sequences using a custom Python script based on the different primer sequences used for 16S and ITS amplification. The R package “DADA2” was then used to generate the amplicon sequence variants (ASVs) table (Callahan et al., 2016). Through “DADA2,” the demultiplexed “fastq” files for each sample were filtered, trimmed and dereplicated to discern the error rates. Forward/reverse reads were merged together, and chimeras were removed from the whole set. The ASVs table was generated and sequences were then assigned to taxonomy through DADA2. “SILVA reference database” (version 132) (Wang et al., 2007; Quast et al., 2012)^3^ was used for 16S amplicon data “assignTaxonomy” function. For fungal taxonomy, the general “fasta” release files from “UNITE ITS database” was used (Version 18.11.2018)^4^. Singletons were removed before subsequent analysis.

### Data Exploration and Statistical Analysis

Based on output from the “DADA2” package, statistical analysis was performed using different R packages (R version 3.5.2)^5^. “VennDiagram” package was used to show the distribution of unique ASVs among different samples (Schwenk, 1984). Alpha-diversity analysis was conducted using “alpha” function from R package “microbiome” (Lahti and Shetty, 2018). Different index value of alpha diversity was obtained while Shannon, Chao1 and InverseSimpson index were plotted through “ggplot2” (Wickham, 2016) -based R package “ggpubr” (Kassambara, 2018). Function “stat_compare_means” from “ggpubr” was used for *T*-test between groups. “Ordinate” function from package “Phyloseq” was used for the Principal coordinate analysis (PCoA) and default distance Bray was applied. “Plot_ordination” function from package “ggplot2” (Wickham, 2016) was used to build the plot. For the summarization of samples taxonomic composition, microbial genomics module of QIAGEN CLC Genomics Workbench 20.0^6^ was used to build the sunburst figures. Taxa with at least 1% of the total reads were then extracted and used to summarize the distribution of taxa across different tissue compartments using R package “Phyloseq” (McMurdie and Holmes, 2013). Package “ggplot2” was used for bar chart plotting. Function “subset_taxa,” “get_taxa” and “sample_sums” from package “Phyloseq” were used to extract taxa of interest and get read abundance from taxa of interest as well as sample of interest. Unpaired *T*-test and ANOVA analysis were carried out to compare taxa abundance among groups using Prism Graphpad software^7^. For further insight into the microbial distribution pattern across rice tissue compartments, data from seeds and seedlings were combined and taxa presenting more than 0.1% of the total reads were extracted, normalized and subjected to K-means clustering. The distance matrices were made by using the “vegdist” function in R package “Vegan” (Oksanen, 2015) and the clusters were then generated by hierarchical agglomerative clustering (function “hclust”) using complete linkage. This multivariate clustering analysis was used to reveal similar groupings of taxa as cluster patterns in the dataset across tissues. The taxa included in these clusters are shown in Supplementary Tables 5, 6. In the end, core members of the microbial communities were extracted using R package microbiome (Lahti and Shetty, 2018) with 100% representation (i.e., present in all 6 samples within a group, seedling samples were grouped based on the 4 compartments). When compare seedlings data with previous seeds data (Eyre et al., 2019), ASV table from seedlings data was combined with ASV table from early published seeds data and then subjected to corresponding analysis.

## Results

### Changes of Microbial Members in the Rice During Shoot and Root Growth

The number of reads before and after quality control and the number of ASVs per sample as well as per tissue compartment are shown in Supplementary Tables 1, 2. After quality control, 18,308,731 total raw reads were separated, trimmed and filtered to yield 4,101,915 bacterial reads and 5,917,486 fungal reads, respectively. With the exception of the root surface sample from fungi, the number of high-quality reads per tissue compartment after quality control ranged between 955,602–2,496,917.

**FIGURE 1:**
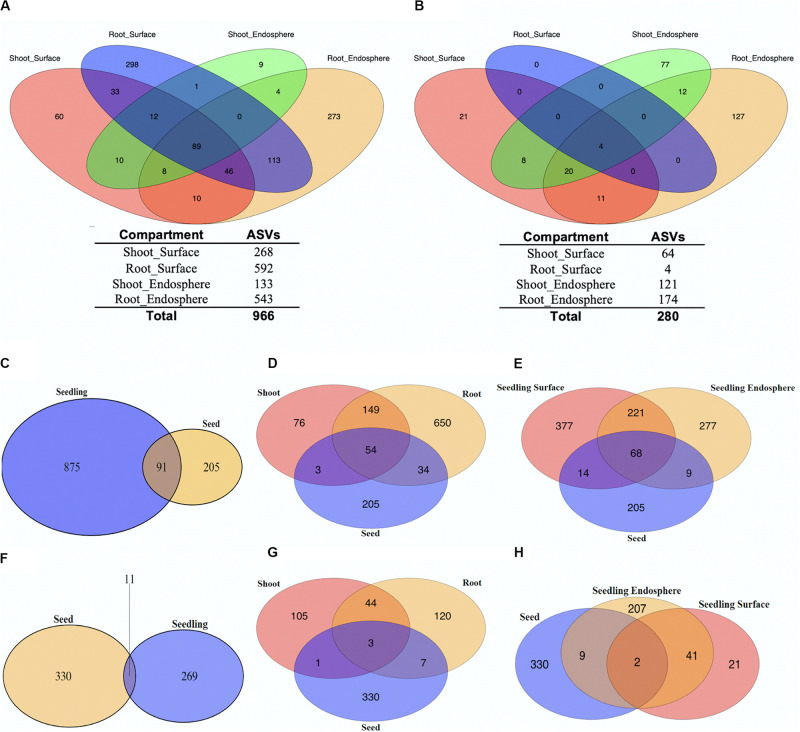
Relationships between unique ASVs by tissue and tissue compartments. Distribution of unique bacterial **(A)** and fungal **(B)** ASVs separated by seedling compartment. The number of unique ASVs found in each seedling compartment are shown below the Venn Diagram (Pink: Shoot_Surface; Blue: Root_Surface; Green: Shoot_Endosphere; Yellow: Root_Endosphere). Unique ASVs in seedlings compared to unique ASVs in seeds **(C–H)**. Distribution of unique bacterial **(C–E)** and fungal **(F–H)** ASVs separated by tissue compartments.

Distribution of unique ASVs as having more than one read in any of seedling tissue compartments was summarized firstly to reveal a broad picture of the microbial members within the rice. The Venn diagrams shown in Figures 1A,C–E showed the distribution of bacterial members within different rice seedling compartments (shoot_endosphere, shoot_surface, root_endosphere, and root_surface). Examination of the 4 seedling compartments revealed a total of 966 unique ASVs (Figure 1A). More ASVs were found in root tissue (887) than in shoots (282). For both the root and shoot tissues, the number of ASVs was slightly higher in the surface samples (680) compared to the endosphere (575). In addition, for both the surface and endosphere sample, the root contained more ASVs than shoot samples (Root surface: 592 vs. Shoot_surface: 268; Root endosphere: 543 vs. Shoot endosphere 133). Overall, 640 (66.3%) of the ASVs were uniquely found only in single seedling compartments: 298 (30.8%) out of all ASVs were only found in root surface sample; 273 (28.3%) for the root endosphere; 60 (6.2%) for the shoot surface and 9 (0.9%) for the shoot endosphere. Of the total 966 microbial ASVs, only 89 (9.2%) were shared by all 4 seedling compartments.

Based on our previously published data (Eyre et al., 2019), a total of 296 ASVs were detected in the rice seeds. Comparison of the rice seeds and seedling data sets revealed 91 ASVs were shared, representing 30.7% of those present in the seeds (7.8% of total ASVs) as shown in Figure 1C. When the seedling data sets were separated into shoots and roots, 54 ASVs were shared by rice seeds, shoots and roots (Figure 1D). However, 88 of the 91 were shared between roots and seeds, whereas 57 of the 91 were shared between shoots and seeds. On the other hand, the shared ASVs only represented 9.9% (88 out of 887) of root ASVs while representing 20.2% (57 out of 282) of shoot ASVs. Seedling samples were further separated to seedling surface and seedling endosphere. Inspection revealed that 68 ASVs were shared between rice seeds, seedling surface and seedling endosphere samples, whereas 82 and 77 of seed ASVs were shared with seedling surface and seedling endosphere samples, respectively. The shared ASVs accounted for 12.1% (82 out of 680) of total seedling surface ASVs and for seedling endosphere, the shared ASVs account for 13.4% (77 out of 575). In sum, from the perspective of the seed, a greater number of the bacterial seed microbiome was retained by the root than the shoot, but these seed derived microbes showed little preference for being retained in the seedling surface or endosphere compartments.

To better understand the microbiome dynamics from seeds to seedlings, additional analyses were performed using the four different seed compartments: outer husk, husk, outer grain and grain (Eyre et al., 2019; see Supplementary Figure 1). From outer surface to inner grain, the number of shared ASVs among seed compartments and seedling samples decreased, consistent with the observation that the number of ASVs decreased in rice seeds from outer surface to inner grain (Eyre et al., 2019). From the perspective of seed compartments, 43.6% (85/195) of outer husk ASVs were shared with seedling samples (roots and shoots); 39.0% (57 out of 146) of husk ASVs were shared with seedlings; 41.2% (35 out of 85) of outer grain ASVs were shared while only 18.9% (7 out of 37) of grain ASVs were shared (Supplementary Figures 1A–D). Similar patterns were observed when comparing seed compartments with seedling surface and endosphere compartments (Supplementary Figures 1E–H). Thus, overall, although the outer husk contributed the most ASVs to the seedling microbiome, there appeared to be little preference based on proportion regarding which seed compartment contributed predominantly to the seedling microbiome, with the possible exception of the grain which contributed the fewest and lowest proportion.

For the fungal dataset, 280 ASVs in total were detected for rice seedlings (Figure 1B). Overall, 225 (80.4%) of the ASVs were found to be uniquely associated with specific seedling compartments: 21 (7.5%) ASVs were only found in shoot surface sample; 0 (0%) for root surface; 77 (27.5%) were found specific for shoot endosphere and 127 (45.4%) for root endosphere. Only 4 of the total 280 ASVs were shared by all 4 seedling samples, all of which were found on the root surface. The low number of ASVs found on the root surface preclude any further general inferences regarding the effect of organs (root/shoot) and location (surface/endosphere) impacting the fungal communities.

Seedling fungal data were then compared with previous rice seeds data (Eyre et al., 2019) where 341 fungal ASVs were detected (Figures 1F–H). Only 11 ASVs were shared, representing 1.8% of the total ASVs (3.2% of seed data set). Similar to the bacterial analysis, seedling samples were then separated by shoots and roots: 3 ASVs which represents 0.5% of total were shared by rice seeds, shoots and roots. During germination, 10 [out of 341 (2.9%)] of seeds ASVs were shared with root samples while 4 [out of 341 (1.2%)] of seeds ASVs were shared with shoot samples. Moreover, the shared ASVs represented 5.8% (10 out of 174) of root ASVs and represented 2.6% (4 out of 153) of shoot ASVs. Seedling samples were further separated to seedling surface and seedling endosphere. Only 2 ASVs which represent 0.3% of total were shared by rice seeds, seedling surface and endosphere samples. During germination, the 11 [out of 341 (3.2%)] seed ASVs were shared with seedling endosphere samples while only 2 [out of 341 (0.6%)] were shared with seedling surface samples. The shared ASVs accounted for 4.2% (11 out of 259) of total seedling endosphere ASVs. For the seedling surface, the shared ASVs accounted for 3.1% (2 out of 64). Additional analyses were conducted using the four seed compartments: outer husk, husk, outer grain and grain to better understand the microbiome shift from seeds to seedlings (Supplementary Figure 2). From the outer surface to inner interior, 2.7% (7 out of 262) of outer husk ASVs were shared with seedling samples; 3.6% (4 out of 112) of husk ASVs were shared with seedlings; 3.4% (7 out of 211) of outer grain ASVs were shared and 7.6% (5 out of 66) of grain ASVs were shared. Overall, even though the number of fungal ASVs commonly associated with seeds and seedlings was low, each seed compartment contributed fairly evenly to the seedling microbiome, which were predominantly located in the root and endophyte tissues.

### Diversity and Driving Factors of Microbial Communities

**FIGURE 2:**
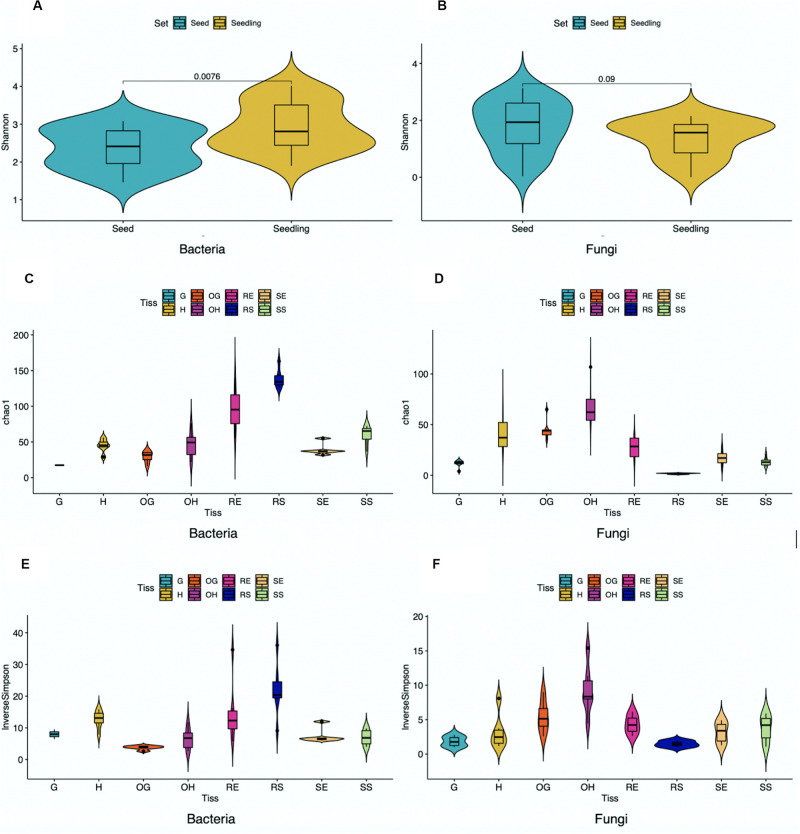
Alpha diversity of bacterial **(A,C,E)** and fungal **(B,D,F)** ASVs within samples pooled based on tissue compartments. Unpaired *t*-test was performed, and *P*-values were added in **(A,B)** (G,Grain; H, Husk; OG, Outer Grain; OH, Outer Husk; RE, Root Endosphere; RS, Root Surface; SE, Shoot Endosphere; SS, Shoot Surface).

To evaluate diversity of microbial communities associated with rice seedlings, alpha diversity was calculated across samples grouped to different compartments, years, genotypes and locations (Figure 2 and Supplementary Tables 3, 4). Alpha diversity provides information regarding species richness (ASV abundance) and diversity within single samples. For rice seedlings, associated bacteria were more diverse than associated fungi. Moreover, root samples were more diverse than shoot samples while the surface samples were more diverse than the endosphere samples (except for fungi associated with shoot surface compartment). Analysis of the combined seeds data with seedlings data indicated that bacteria associated with seedlings were more diverse than those associated with seeds while fungi associated with seed samples were slightly more diverse than those associated with seedlings. Other factors including genotype, location and year also had minimal effect on diversity.

**FIGURE 3:**
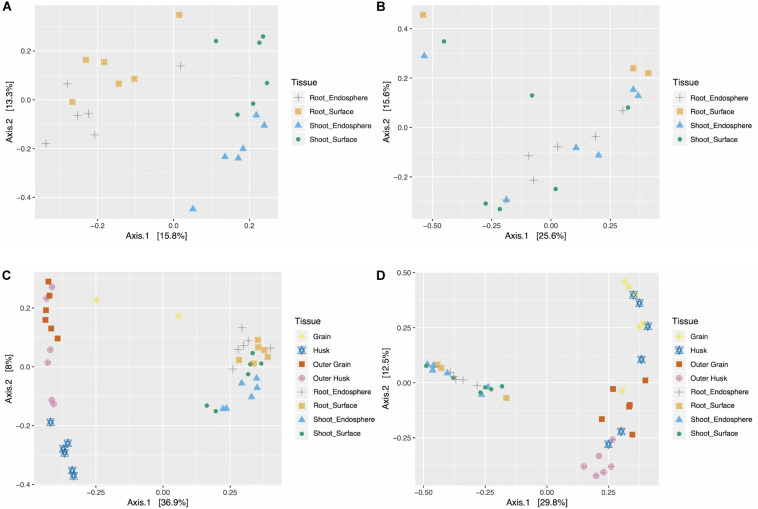
Bacterial and fungal Principal coordinate analysis (PCoA) for seedlings **(A,B)** and combined data (seeds and seedlings data) **(C,D)**. PCoA was performed on the bacterial **(A,C)** and fungal **(B,D)** samples, colorized by different tissue compartment (G,Grain; H, Husk; OG, Outer Grain; OH, Outer Husk; RE, Root Endosphere; RS, Root Surface; SE, Shoot Endosphere; SS, Shoot Surface).

To better understand the impact of different factors (seedling compartment, harvesting year, harvesting location, rice genotype) on the microbial community, Principal Coordinates Analysis (PCoA) was used to explore the internal relationships of those variables (Figures 3A,B). For bacterial and fungal datasets of rice seedlings, PCoA plots showed that samples generally clustered together based on different tissue compartments, indicating distinct communities. However, when samples were grouped based on different harvesting year, location or rice genotype, no obvious clusters were evident (Supplementary Figures 5, 6). Seeds data were also combined with seedling data and subjected to PCoA analysis (Figures 3C,D). Microbiome community (both bacterial and fungal) associated with rice seeds were very distinct from those associated with seedlings. For rice seeds, consistent with previous publication (Eyre et al., 2019), the grain compartment formed the most distinct bacterial grouping. Inspection of the fungal PCoA in seeds samples revealed that the grain, outer grain, and outer husk tissues formed distinct groupings with the husk overlapping all three. Though the seedling sample did not show clear community patterns in the combined analysis with the seed data, when the seed data was removed, the bacterial community possessed by shoot samples was different from root samples and the microbiome associated with plant surface was distinct from the plant endosphere (as shown in Figures 3C,D).

### Taxon Composition of Microbial Communities

**FIGURE 4:**
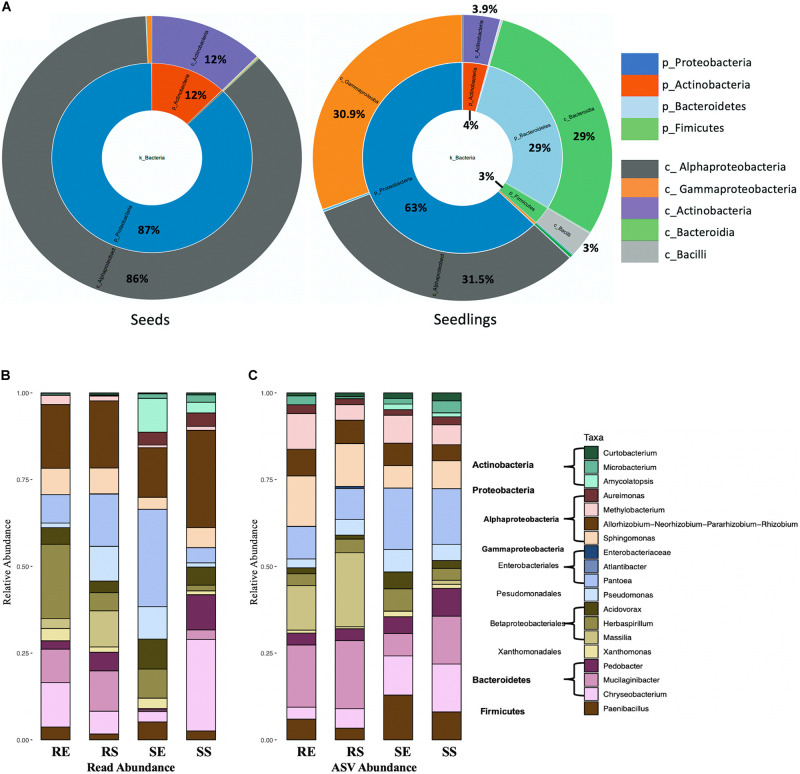
Bacterial taxon composition for microbial communities. Comparison of seeds and seedlings data at phylum and order level **(A)**. Bacterial genera bar graphs based on reads abundance **(B)** and ASVs abundance **(C)** for 4 different tissue compartments (Top genera with > 1% total reads). Tissue compartment: RE, Root Endosphere; RS, Root Surface; SE, Shoot Endosphere; SS, Shoot Surface.

To better understand changes in microbial communities during germination, CLC workbench (Microbial genomics module) was used to visualize taxa proportions for comparing seeds and seedlings. For bacteria (Figure 4A and Supplementary Figure 3), Proteobacteria (87%) and Actinobacteria (12%) composed the entire seeds bacterial community. Though Proteobacteria were also dominant for seedlings (63%), reduced Actinobacteria (4%) were detected along with emerging Bacteroidetes (29%) and Firmicutes (3%). In addition, during germination the abundance of Gammaproteobacteria increased compared to seeds (from 0.9 to 30.9%, *P* = 0.0259) where Alphaproteobacteria were prevalent (86% in seeds).

A total of 247 taxonomic classifications primarily at the genus level were detected for the combined seed and the seedling datasets. Nineteen taxa were identified representing 91.5% of the total reads (Figure 4B, seeds data and seedlings data combined). Of those taxa, 12 were from proteobacteria (4 Alphaproteobacteria and 8 Gammaproteobacteria), 3 were from Bacteroidetes, 3 Actinobacteria and 1 Firmicutes. Considering the seedling samples, 16 taxa were present in the combined data set (taxa *Curtobacterium*, *Microbacterium*, *Enterobacteriaceae*, and *Atlantibacter* were absent from seedlings while *Luteibacter* was included). Taxon composition of the root endosphere was similar as the root surface, except for increased abundance of *Pseudomonas* (from 1.3 to 10%, *P* = 0.0028), *Massilia* (from 2.8 to 10.4%, *P* = 0.0206) and reduced abundance of *Herbaspirillum* (from 21.4 to 5.2%, P = 0.0250). The shoot sample contained a greater abundance of Actinobacteria (8.5% for shoot and 0.6% for root, *P* = 0.0009) and *Aureimonas* (3.8% for shoot and 0.2% for root, *P* = 0.001) than roots. The shoot endosphere sample was richest in *Pantoea* and least rich for *Bacteroidetes*. In contrast, in the shoot surface sample, Gammaproteobacteria (from 57.4 to 13.6%, *P* < 0.0001) were reduced while Bacteroidetes (from 3.8 to 39.3%, *P* < 0.0001) increased. When examined based on ASV abundance distribution (Figure 4C) rather than read abundance, compared to read abundance bar plot, *Methylobacterium* increased in seedlings and *Actinobacteria* increased in root samples. For the shoot endosphere sample, *Bacteroidetes* ASVs were highly prominent.

With respect to seeds, 8 taxa were included in the 19 taxa in the combined seeds and seedlings dataset. These observations were similar to previous findings using the seeds data alone, where 9 taxa were identified whose abundance were higher than 1% of total reads with the addition of *Franconibactor* (Eyre et al., 2019). Moreover, the taxon composition in rice seedlings was distinct from seed samples. *Curtobacterium* and *Microbacterium* from Actinobacteria were consistently present for all tissue compartments, however, the abundance was reduced in seedling samples compared to rice seed (*Curtobacterium* from 8 to 0.4%, *P* < 0.0001; Microbacterium from 2 to 1%, *P* = 0.0281). A similar pattern was also observed for 4 genera from Alphaproteobacteria, which were very prominent in seeds. In contrast, compared to seed samples, members from Gammaproteobacteria, Bacteroidia and Firmucutes were abundant in seedlings and represented 47.4% of the total reads.

**FIGURE 5:**
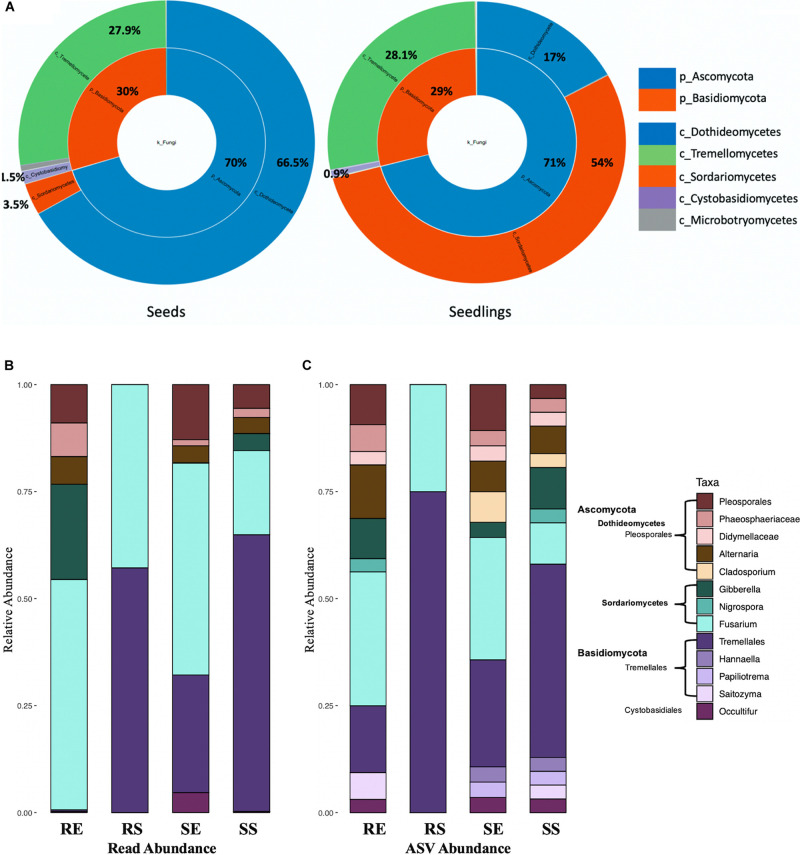
Fungal taxon composition for microbial communities. Comparison of seeds and seedlings data at phylum and order level **(A)**. Fungal genera bar graphs based on reads abundance **(B)** and ASVs abundance **(C)** for 4 different tissue compartments (Top genera with > 1% total reads). Tissue compartment: RE, Root Endosphere; RS, Root Surface; SE, Shoot Endosphere; SS, Shoot Surface.

For fungi, the seed and seedling communities were comprised of Ascomycota and Basidiomycota (Figure 5A). Tremellomycetes (from 27.9 to 28.13%, *P* > 0.05) and Cytobasidiomycetes (from 1.5 to 0.9%, *P* > 0.05) were the most abundant taxa for Basidiomycota and their total proportion remained unchanged during germination (for seedling samples, 99% ASVs from Tremellomycetes could not be assigned to a specific genus while in Cytobasidiomycetes, genus *Occultifur* emerged to be dominant as the genus *Symmetrospora* became undetectable). In contrast, for Ascomycota, the abundance of Sordariomycetes increased dramatically (from 3.5 to 54%, *P* < 0.0001) while Dothideomycetes were reduced (from 66.5 to 17%, *P* < 0.0001) in seedlings. It should also be noted that for Sordariomycetes, *Fusarium* became prevalent in seedlings compared to seeds where *Nigrospora* was the primary (Supplementary Figure 4).

In the fungal data, 159 taxonomic classifications primarily at the genus level were detected for rice seedlings. Similar to bacteria, fungal taxa with at least 1% of the reads (13 genera representing 90.6% of total reads) from seeds and seedlings dataset were examined (Figure 5B, combined seeds and seedlings dataset). Overall, taxa assigned to *Fusarium* and *Tremellales* accounted for 74.8% (Figure 5B) of the whole seedling taxon composition. However, compared to other seedling samples, Tremellales (0.5% for root endosphere, ANOVA *P* = 0.0024) and all Basidiomycota (0.6% for root endosphere, ANOVA *P* = 0.0021) were poorly represented in the root endosphere compartment (Figure 5B). Inspection based on ASV abundance rather than read abundance, revealed that *Occultifur* which only represented 0.15 and 0.04% of reads in root endosphere and shoot surface compartments showed higher relative ASV abundance. Furthermore, in the root endosphere, based on reads Basidiomycota accounted for less than 2% of the root endosphere reads, whereas they accounted for ∼25% of ASV abundance (Figure 5C).

With regards to changes in fungal taxa during seedling development, taxa *Pleosporales* (from 42.3 to 7.9%, *p* < 0.0001), Didymellaceae (from 11.1% to non-detectable), *Alternaria* (from 13.9 to 4%, *P* = 0.0072) and *Cladosporium* (from 4.5% to non-detectable) diminished while *Fusarium* (from non-detectable to 41.6%) and Tremellales (from 4.8 to 34.2%, *P* = 0.0002) increased. Although only 4 fungal ASVs were found on the root surface they were predominantly Tremellales. Taxa such as *Papiliotrema*, *Saitozyma*, *Hannaella*, *Nigrospora*, *Gladosporium* and Didymellaceae showed increased relative ASV abundance when compared to read abundance (Figures 5B,C).

### Microbiome Patterns Across Rice Tissue Compartments

**FIGURE 6:**
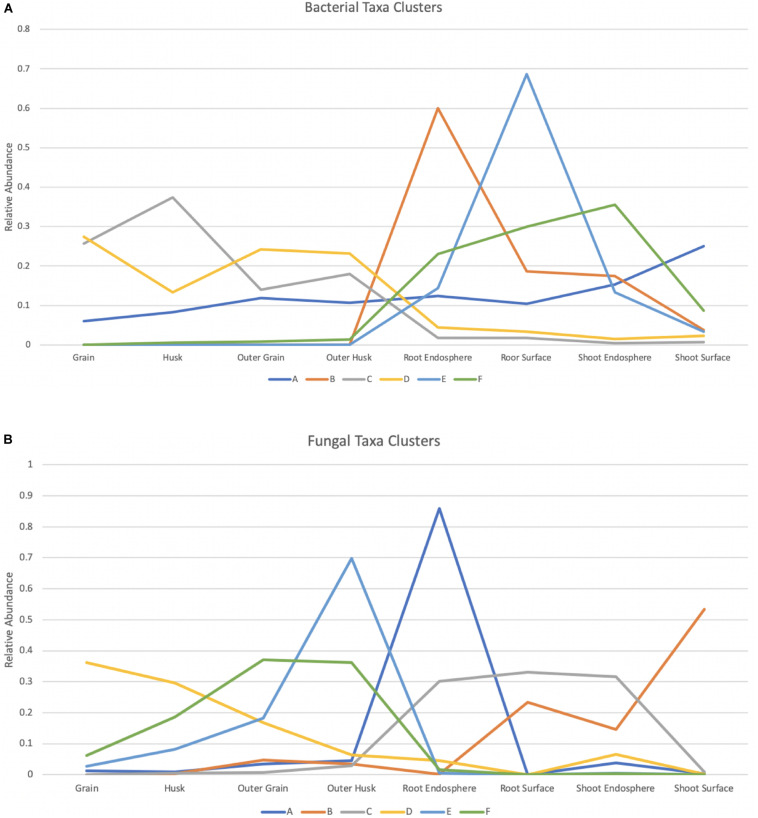
Bacterial and fungal taxa clusters (Microbial taxa with > 0.1% total read abundance). Clustering of the normalized relative abundance values for bacterial **(A)** and fungal **(B)** taxa. Node values represent the average of the normalized abundance values within a cluster for each of the tissue compartments, A–F represent the 6 clusters summarized from the data and taxa included in each cluster can be found in Supplementary Tables 5, 6.

Multivariate clustering analysis showed the bacterial data was assigned to 6 clusters across tissue compartments (Figure 6A and Supplementary Figure 7). Taxa assigned to cluster B were found predominately in the root endosphere which contained 28.6% of the total root endosphere reads. Members of this cluster were primarily from the Proteobacteria. Taxa in cluster E were abundant in root surface compartments representing 20.1% of the root surface reads. Members of clusters B, E, and F, which were prominent in seedlings, were largely absent from seeds. Cluster A, which was made up of 10 taxa including *Rhizobium*, *Paenibacillus*, *Pedobacter*, and *Microbacterium*, was prominent in both seed and seedling compartments. Taxa in cluster C were dominant in seeds, particularly in grain and husk samples and included taxa *Cautobacterium*, *Kineococcus* as well as *Methylobacterium*. Similar to Cluster C, Cluster D also contained taxa dominant in seeds compartments such as *Brevundimonas*, *Sphingomonas*, and *Roseomonas*.

K-means clustering was also applied to the fungal dataset and provided 6 clusters across 8 tissue compartments (Figure 6B and Supplementary Figure 7). Taxa in cluster A were dominant in the root endosphere sample and were all Ascomycota. They represented 34.5% of total ASV reads in this compartment. Six taxa in cluster E including *Saitozyma* and *Nigrospora* were found mostly in outer husk compartment representing 33.7% of outer husk reads. Half of them were Ascomycota while the other half were Basidiomycota. Cluster C (*Occultifur* and *Fusarium* included) contained taxa that were in high abundance in the root samples and shoot endosphere compartment. They were largely absent from seeds. A similar pattern was found for Cluster B, which contained taxa absent from seeds but abundant for root surface and shoot samples. Furthermore, this cluster had modest representation in the outer husk and outer grain compartments of seeds. Only one taxon was present in Cluster B: Tremellales. Cluster F was primarily restricted to seeds and carried taxa that were most abundant in outer grain and outer husk compartment such as *Hannaella* and *Phaeosphaeria*. Of note, members of Cluster D, which included *Alternaria* and *Curvularia* taxa were detected in all seed compartments but highest in grain.

### Identification of a Core Microbiome

In total, 25 bacterial taxa and 8 fungal taxa were identified as core members in one or more seedling compartments. The bacterial core represented 90.0% of the bacterial total reads, while the fungal core represented 61.3% of the fungal total reads. From the perspective of ASVs, the ASVs identified in the bacterial core represented 42.2% (494/1171) of the bacterial ASVs, while the fungal core represented 21.8% (133/610) of the fungal ASVs.

Considering the bacterial core (Table 1), 10 taxa were detected in all samples of the root endosphere; 21 taxa for root surface samples; 11 taxa for shoot endosphere samples and 15 for shoot surface. Genera including *Allorhizobium*, *Sphingomonas, Methylobacterium, Aureimonas, Pantoea*, and *Xanthomonas* were consistently detected as core for all four tissue compartments. Less prevalent taxa such as *Microbacteriaceae* and *Rhizobiaceae* were only absent for root endosphere samples; *Mucilaginibacter* and *Paenibacillus* were consistently detected as core except for shoot endosphere samples. *Curtobacterium*, *Pseudomonas*, and *Chryseobacterium* were only identified in surface samples.

**TABLE 1:** Bacterial core seedling microbiome.

	RE	RS	SE	SS
Actinobacteria		Microbacterium	Amycolatopsis	Amycolatopsis
		(−, **1**, 1, 1)	(−, −, **1**, 1)	(−, −, 1, **1**)
		Microbacteriaceae	Microbacterium	Microbacterium
		(−, **1**, 1, 1)	(−, 1, **1**, 1)	(−, 1, 1, **1**)
		Curtobacterium	Microbacteriaceae	Microbacteriaceae
		(−, **1**, −, −)	(−, 1, **1**, 1)	(−, 1, 1, **1**)
		Kineococcus		Curtobacterium
		(−, **1**, −, −)		(−, −, −, **1**)
		Quadrisphaera		
		(−, **1**, −, −)		
Proteobacteria	Allorhizobium	Allorhizobium	Allorhizobium	Allorhizobium
Alphaproteobacteria	(**2**, 2, 1, 1)	(2, **2**, 1, 1)	(1, 1, **1**, 1)	(1, 1, 1, **1**)
	Sphingomonas	Sphingomonas	Sphingomonas	Sphingomonas
	(**4**, 2, 1, 1)	(2, **7**, 2, 2)	(1, 2, **2**, 2)	(1, 2, 2, **2**)
	Methylobacterium	Aureimonas	Aureimonas	Aureimonas
	(**4**, 2, 1, 1)	(−, **2**, 1, 1)	(−, 1, **1**, 1)	(−, 1, 1, **1**)
	Novosphingobium	Rhizobiaceae	Rhizobiaceae	Rhizobiaceae
	(**1**, 1, −, −)	(−, **1**, 1, 1)	(−, 1, **1**, 1)	(−, 1, 1, **1**)
	Aureimonas	Methylobacterium	Methylobacterium	Methylobacterium
	(**1**, −, −, −)	(2, **3**, 1, 1)	(1, 1, **1**, 1)	(2, 1, 1, **2**)
		Novosphingobium		
		(1, **1**, −, −)		
		Roseomonas		
		(−, **2**, −, −)		
		Belnapia		
		(−, **1**, −, −)		
Proteobacteria	Pantoea	Pantoea	Pantoea	Pantoea
Gammaproteobacteria	(**2**, 2, 1, 1)	(2, **6**, 1, 1)	(1, 1, **1**, 1)	(1, 1, 1, **1**)
	Xanthomonas	Luteibacter	Xanthomonas	Xanthomonas
	(**1**, 1, 1, 1)	(−, **1**, −, −)	(1, 1, **1**, 1)	(1, 1, 1, **1**)
	Herbaspirillum	Pseudomonas	Cupriavidus	Pseudomonas
	(**1**, −, −, −)	(−, **3**, −, 2)	(−, −, **1**, −)	(−, 2, −, **2**)
		Massilia		
		(−, **1**, −, −)		
		Xanthomonas		
		(1, **1**, 1, 1)		
Bacteroidetes	Mucilaginibacter	Chryseobacterium		Chryseobacterium
	(**3**, 2, −, 1)	(−, **1**, −, −)		(−, −, −, **1**)
		Mucilaginibacter		Mucilaginibacter
		(2, **3**, −, 1)		(1, 1, −, **1**)
Firmicutes	Paenibacillus	Paenibacillus		Paenibacillus
	(**1**, 1, −, 1)	(1, **3**, −, 1)		(1, 1, −, **1**)

*The bacterial ASVs and their representative taxa shared between all samples of a seedling compartment. The numbers in parentheses represent the number of ASVs belonging to the bacterial taxa that are shared with other compartments in order according to the header. The bold number represents the number of ASVs belonging to the compartment of interest (RE, Root Endosphere; RS, Root Surface; SE, Shoot Endosphere; SS, Shoot Surface).*

For the fungal core (Table 2), 7 taxa were detected in all samples of the root endosphere; 1 taxon for root surface samples; 3 taxa for shoot endosphere samples and 2 for shoot surface. *Fusarium* was consistently detected except for the shoot surface samples and Pleosporales was consistently detected except for the root surface samples. *Alternaria* was only detected in the endosphere sample. For the Basidiomycota, only Occultifur in the root endosphere and Ustilaginaceae in the shoot surface were found. Ascomycota such as Didymellaceae, Phaeosphaeriaceae and *Clonostachys* were also identified as core for the root endosphere.

**TABLE 2:** Fungal core seedling microbiome.

	RE	RS	SE	SS
Ascomycota	Pleosporales		Pleosporales	Pleosporales
Dothideomycetes	(**1**, −, 1, 1)		(1, −, **2**, 1)	(1, −, 2, **1**)
	Alternaria		Alternaria	
	(**2**, −, 1, −)		(2, −, **1**, −)	
	Didymellaceae			
	(**1**, −, −, −)			
	Phaeosphaeriaceae			
	(**1**, −, −, −)			
Ascomycota Sordariomycetes	Fusarium	Fusarium	Fusarium	
	(**1**, 1, 1, −)	(1, **1**, 1, −)	(1, 1, **2**, −)	
	Clonostachys			
	(**1**, −, −, −)			
Basidiomycota	Occultifur			Ustilaginaceae
	(**1**, −, −, −)			(−, −, −, **1**)

*The fungal ASVs and their representative taxa shared between all samples of a seedling compartment. The numbers in parentheses represent the number of ASVs belonging to the bacterial taxa that are shared with other compartments in order according to the header. The bold number represents the number of ASVs belonging to the compartment of interest (RE, Root Endosphere; RS, Root Surface; SE, Shoot Endosphere; SS, Shoot Surface).*

Core microbiome found in seedlings were compared to those found in seed samples (Eyre et al., 2019). Generally, the seed bacteria core was a subset of the seedling except for Franconibacter found in grain. Genera of *Methylobacterium, Aureimonas, Rhizob*ium and *Sphingomonas* were consistently detected in seed and seedling samples. However, for the fungal core, genera contained in seedlings were a subset of those contained in seed samples except for *Clonostachys* and Ustilaginaceae. Pleosporales, *Alternaria*, Didymellaceae, Phaeosphaeriaceae and Occultifur were dominant in seed samples while *Fusarium* was only detected as core in the outer husk.

## Discussion

Microbes can colonize different plant compartments and prosper on the outer surfaces as well as inside plant tissues (Turner et al., 2013; Berg et al., 2014). In this study, we first explored the microbiome associated with rice seedlings derived exclusively from seeds. For the seedling bacterial data, fewer ASVs were detected in endosphere samples than surface samples. This may due to physical as well as biochemical barriers that restrict microbes from colonizing inside plants. In addition, the roots harbored more ASVs than shoots, which may be a result of the soil facilitating microbial growth within roots. However, a similar pattern was not seen in the fungal data where limited ASVs were detected in the root surface sample. Perhaps the method of sample collection of roots, which involved gentle shaking to remove debris and the method used for amplicon production may explain the small number of ASVs in fungal data. Overall, the percentage of shared ASVs in the 4 seedling compartments was low both for fungi and bacteria, suggesting tissue compartment as the driving factor of microbial communities. The PCoA analysis further confirmed this conclusion.

When compared to the previous seed data (Eyre et al., 2019), shared bacterial as well as fungal ASVs constituted a low proportion of the whole, indicating ASV composition in the seeding is very different from seeds and development plays an important role in proliferation of the rice associated microbiomes. This was evident from the PCoA analysis. The high proportion of seedling specific bacterial and fungal ASVs may due to the nutrient rich environment provided by soil and/or nutrients released from seedlings during germination. As such, rare microbes, possibly existing as fungal and bacterial spores in the seed prospered in the seedlings and were identified as unique ASVs (Darrasse et al., 2010; Huang et al., 2016; Johnston-monje et al., 2016; Shade et al., 2017). However, it is also formally possible that the seedling ASVs from sand result from DNA contamination, present even in sterile sand.

For the bacterial microbiome, the ASV pool of the grain contributed the least ASVs to the seedling while the outer husk contributed the most. This may imply some valuable function associated with the outer husk compartment, whereby microbes are recruited from the parent plant during growth and development and may confer some benefit to rice growth. When the seeds germinate, those microbiome from the outer husk are thus recruited again to favor rice seedlings. Microbes from the grain compartment on the other hand, may be highly specialized and do not thrive as robustly as epiphytes during seedling growth due to unsuitable living environment and resource limitations (James et al., 2002; Compant et al., 2010; Turner et al., 2013).

The total number of fungal ASVs of seeds and seedlings were similar. However, the amount of shared ASVs between seeds and seedlings were extremely low. This may be result of the methods used for sample collection, amplicon production and two independent sequencing data processing for seed and seeding. Alternatively, germination and development play a major role in establishment of the seedling fungal community. Many fungi found in the seeds may be opportunistic saprophytes and are readily lost and fall to levels below our limits of detection during seedling growth (Afkhami and Rudgers, 2008; Márquez et al., 2012). Nevertheless, the outer husk and outer grain compartments contributed the most fungal ASVs to the seedling which may be due to the high diversity of fungi associated with those two compartments (Figure 2).

Different field conditions and agricultural activities alter the microbial community (Buyer et al., 1999; Hacquard, 2016), as may genetic differences of host plants (Peiffer et al., 2013). In this study, it was the tissue compartment that proved to be the principal driving factor of microbial community. This discovery also suggested that there may be core microbiome consistently associating with rice plants regardless of location, genotype and harvesting time. Additional studies using rice representing more diverse genotypes from more growing locations and harvesting years would be needed to confirm conclusions obtained in this study. In fact, little is known about the mechanisms for microbial community build up. More knowledge is needed regarding the interaction between host and microbiome as well as interaction among different microbial communities (Lau and Lennon, 2011; Cordero and Datta, 2016; Henry et al., 2016).

Taxa composition of tissue compartments revealed here are consistent with previous studies related to microbiome communities associated with plants (Fischer et al., 2012; Lundberg et al., 2012; Sessitsch et al., 2012; Vorholt, 2012; Bodenhausen et al., 2013; Bulgarelli et al., 2013; Schlaeppi and Bulgarelli, 2015). Similar taxon compositions were detected in the root endosphere and on the root surface, indicating that both of those two compartments inherited similar microbial taxa from seeds. However, some differences were noted, indicating that the endosphere may impose some selection mechanisms. More *Pseudomonas* and *Massilia* accumulated in root endosphere rather than on the root surface and those microbes are strongly linked to plant growth promotion. It is noteworthy that ASV abundance was also analyzed in addition to read abundance. In a number of instances, taxa showed dramatic differences in read abundance compared to their taxonomic (ASV) abundance. For those who had lower proportion of read abundance but higher ASV abundance such as *Actinobacteria* in roots, it may suggest a higher evolution potential for this specific taxon. On the contrary, for the *Rhizobium* genus from Alphaproteobacteria, ASV abundance in seedlings was lower than read abundance, suggesting ASVs detected in this genus are quite conservative.

A point worth highlighting is that though not detected in seeds, Bacteroidetes (29%) and Firmicutes (3%) were detected in seedling samples. It is likely that Bacteroidetes and Firmicutes exist in the seed samples in the first place, but the amount of those bacteria fell below our limits of detection in seeds. Previous research had identified Bacteroidetes and Firmicutes associated with rice seeds (Okunishi et al., 2005; Mano et al., 2006; Zhang et al., 2019). The reason they were identified elsewhere may be because they were either isolated bacteria from culturable colonies (Okunishi et al., 2005; Mano et al., 2006) or larger sample amounts for gDNA were used (Zhang et al., 2019). Also, the rice varieties and rice growing conditions were different from our studies, which may have enhanced these taxa in seeds. It is likely that during the process of rice germination, rich nutrients either from rice shoots and roots or soil facilitate the thriving of Bacteroidetes and Firmicutes. Moreover, those bacteria may promote rice growth, generating a mutualism interaction with rice (Urai et al., 2008; Madhaiyan et al., 2010a; Köberl et al., 2011). In fact, considerable microbiome research has revealed a close relationship of rice plants with Bacteroidetes and Firmicutes (Mano and Morisaki, 2008; Raweekul et al., 2016; Lu et al., 2018), consistent with our findings for seedlings.

Proteobacteria, which predominated root endosphere compartments (Cluster B Figure 6A), likely represent specific root endophytes. Other Proteobacteria dominated the root surface (Cluster E), indicating those bacteria live in association with roots and were not selected as endophytes. Cluster A revealed taxa found in the seed that remained in the seedling compartments. This cluster is made up of *Paenibacillus*, *Acidovorax, Pedobacter, Rhizobium, Microbacterium*, and others. It is not known how exactly these taxa are selected, but they may be of particular interest.

From the fungal clustering analysis, taxa were generally found to be associated with specific compartments. Taxa enriched in the root endosphere sample (Cluster A) were identified as Ascomycota. *Gibberella* which can infect rice and produce gibberellin was present in this cluster and gibberellin is a growth hormone promoting cell elongation, flower formation and seedling growth (Cerdá-Olmedo et al., 1994; Zainudin et al., 2008). Furthermore, members of the genus *Clonostachys* found in this cluster have been developed as biological control agents (Xue, 2003; Jensen et al., 2004; Rodríguez et al., 2011).

An important goal of this work was to define a core microbiome of rice for both bacteria and fungi as these may represent microbes that confer beneficial properties. A number of core bacteria were identified, such as *Rhizobium-Allorhizobium*-*Pararhizobium*-*Neorhizobium* that can fix nitrogen and colonize inside plant tissue. These microbes have been also found colonizing roots of non-legume crops such as wheat, barley, maize and rice and could be used as biofertilizer through bio-inoculating with crop seeds (Boddey et al., 1995; Webster et al., 1997; Yanni et al., 1997; Gutierrez-Zamora and Martinez-Romero, 2001; Lupwayi et al., 2004; Chi et al., 2005; Da et al., 2011; Ren et al., 2011; Mousavi et al., 2014, 2015). Species from the genus *Pantoea* (Monier and Lindow, 2005) have been found as part of the epi- and endophyte flora of various plant hosts. They are considered to be phosphate-solubilizing microorganisms (PSMs) and may be valuable to solubilize inorganic phosphates (Son et al., 2006; Coutinho and Venter, 2009). *Pseudomonas, Bacillus*, and *Enterobacter* are also known as PSMs (Raj et al., 1981; Laheurte and Berthelin, 1988). A rice endophyte *Pantoea agglomerans* YS19 was further demonstrated to have nitrogen-fixing activity, producing phytohormones that can improve rice biomass and affect allocations of host photosynthates (Feng et al., 2006). This species was also found to have anti-disease properties that protect pear and apple from *B. cinerea*, *Penicillium expansum*, and *Rhizopus stolonifer* (Nunes et al., 2001; Nunes et al., 2002). Furthermore, *P. agglomerans* may also regulate water content of wheat rhizosphere by improving soil aggregation (Amellal et al., 1998). Siderophores and hydrocyanic acid (HCN) are produced by *Pantoea* which may help with ion absorption and disease control (Selvakumar et al., 2008). However, this genus also contains species that can cause disease on a wide range of host crops as well as human beings (Brenner et al., 1984; Coutinho and Venter, 2009; Kido et al., 2010).

*Sphingomonas*, also detected as a core bacteria occurs in a diverse range of environments, are metabolically flexible and can consume environmental contaminants (Miyauchi et al., 1999; Aylward et al., 2013). Members of this genus can remediate heavy metals and decompose various pesticides (Miller et al., 2010; Liu et al., 2014). *Sphingomonas* sp. LK11 alleviates salinity stress in *Solanum pimpinellifolium* (Khan et al., 2017). *Sphingomonas panaciterrae* sp. nov. was demonstrated to promote plant growth through production of indole-3-acetic acid (IAA) (Sukweenadhi et al., 2015). They were also shown to protect *Arabidopsis thaliana* against bacterial pathogens (Innerebner et al., 2011). Another core bacteria *Paenibacillus* genus have a broad host range and have been demonstrated to have properties such as nitrogen fixation, bioremediation, and promoting plant growth through production of phytohormones including auxin, indole and phenolic compounds. They can also combat plant pathogens and pests by producing antibiotics (Gardener, 2004; Lal and Tabacchioni, 2009; Govindasamy et al., 2010). *P. polymyxa* can enable host drought tolerance (Shiao and Huang, 2001) as well as confer “Induced systemic resistance” (ISR) in *Arabidopsis* through the emission of volatile organic compounds (VOCs) (Lee et al., 2012).

Other core genera, including members of the genus *Mucilaginibacter* are known to have plant-growth-promoting properties and some species have been isolated from dried rice straw in addition to soil samples (Pankratov et al., 2007; Urai et al., 2008; An et al., 2009; Jeon et al., 2009; Luo et al., 2009; Baik et al., 2010; Madhaiyan et al., 2010b). *Methylobacterium* species were shown to promote plant growth through producing different phytohormones and have been isolated from various plants (Kutschera, 2007). They were also known to solubilize calcium phosphate and fix nitrogen (Subhaswaraj et al., 2017). Bacterial species from *Xanthomonas* and *Pseudomonas* may cause plant disease in some circumstances while other species of *Pseudomonas* can also promote plant growth (Cole et al., 2015; Park et al., 2015). Given their known properties, it is likely many of the core bacteria described here have potential to be developed as biologicals for modern agriculture.

Examination of the core fungi associated with rice seedlings, revealed several genera with known biological properties, including members of the *Alternaria* genus. These fungi are ubiquitous in the environment and commonly act as opportunistic plant pathogens (Al-Hatmi et al., 2016). More than 100 plant species can be infected by *Alternaria* species which can cause leaf spot and other diseases (Rotem, 1994). However, some *Alternaria* species also have biocontrol potential against other plant diseases. *A. zinniae, A. eichhornia*, and *A. cassiae* are commercially available for weed control (Walker and Sciumbato, 1979; Walker, 1980; Aneja and Singh, 1989; Babu et al., 2002). *Occultifur* species are basidiomycetous yeasts and usually use plant leaves and soil as important and interrelated habitats (Khunnamwong et al., 2015, 2017). Some species have been reported as mycoparasites, whereas one species has been reported as a saprophyte (Roberts, 1997; Khunnamwong et al., 2015). Members of the family *Didymellaceae* inhabit a wide range of ecosystems (Chen et al., 2017) and most of them are plant pathogens of a wide range of hosts (Aveskamp et al., 2008, 2010; Chen et al., 2015), however, they also comprises several species recognized as endophytic, fungicolous and lichenicolous fungi (Yang et al., 1994; Sullivan and White, 2000; Hawksworth, 2003; Hawksworth and Cole, 2004; Diederich et al., 2007; Schoch et al., 2009). Those core fungi also can be candidates for biocontrol uses.

Filamentous core fungi from the genus *Fusarium* are widely distributed in plants, soil, water and are abundant members of the soil microbial community. Most species are harmless while some species can cause diseases of plants as well as animals. Many products from agriculturally important crops can be contaminated by *Fusarium* spp., which can be of concern because of highly toxic metabolites produced by some species (Rippon, 1982; Walsh and Dixon, 1996; Nowicki et al., 2012). Most species from the order *Pleosporales* are harmless saprobes while there are also species associated with plants as parasites, epiphytes and endophytes (Zhang et al., 2009). The corresponding ASVs could be only assigned to *Pleosporales* at the order level rather than species level, indicating further research is needed to accurately characterize the role of fungi in this order. Fungi from *Gibberella* can infect rice and produce gibberellin, a plant hormone promoting cell elongation, flower formation and seedling growth (Cerdá-Olmedo et al., 1994; Zainudin et al., 2008). *Clonostachys rosea f. rosea* from the *Clonostachys* genus is a plant endophyte and has been used as a biological pest control agent against fungi such as *B. cinerea* as well as nematodes (Toledo et al., 2006; Zhang et al., 2008).

In this research, we identified and characterized the microbiome associated with rice seedlings in a sterile environment. However, the main purpose for this research is to understand the dynamics of microbiota shift from rice seeds to seedlings. Seed-borne microbes is of great interest to researchers because those microbes can be vertically transmitted to next generation (Barret et al., 2015; Cope-Selby et al., 2017; Mitter et al., 2017; Shahzad et al., 2018). During the transmission, phyto-beneficial bacteria and fungi inherited from seeds can promote seedling growth as well as mitigate plant stress damage (Mitter et al., 2017; Shahzad et al., 2018). Knowledge about the microbiota shift from rice seeds to rice seedlings can help uncover what microbes have been transmitted vertically and how well they proliferate. Transmitted microbes showing high abundance in seedlings have great potential to be selected by rice as phyto-beneficial microbes. This will further instruct microbiome inoculant engineering to benefit modern agriculture. The use of a sterile environment to monitor shifts in microbiome populations has been used in other studies (Hardoim et al., 2012; Huang et al., 2016; Mitter et al., 2017; Torres-Cortés et al., 2018). However, there is limited data about how rice seed-borne microbes change during the development process. Our research provides the first detailed description of dynamic microbiota shifts from rice seeds to rice seedlings. Rice seeds of different genotype harvested from different locations at different time allowed us to gain novel insight into these population shifts and the core microbiome associated with seedlings tissue compartments. Further experiments with more varieties and sources of seeds are needed to confirm and extend our findings as well as additional studies to compare population shifts of seeds planted in natural soils.

An initial comparison between our findings and other datasets collected from natural conditions revealed some consistent patterns. Edwards and colleagues (Edwards et al., 2018) found tissue compartments and rice development age were more important factors shaping microbiome than growth location. Wang and colleagues detected more diverse bacterial ASVs in roots than stems while fungal ASVs were more diverse in stems than roots (Wang et al., 2016). Although the identified microbiome varied somewhat between different experimental set ups, similarities in the distribution of phyla are apparent, in line with our key findings. For example, Proteobacteria, Actinobacteria, Bacteroidetes and Firmicutes were consistently detected as bacterial phyla associated with rice while Ascomycota and Basidiomycota were found to be dominant fungal phyla (Mano and Morisaki, 2008; Edwards et al., 2015; Bertani et al., 2016; Raweekul et al., 2016; Venkatachalam et al., 2016; Lu et al., 2018; Thapa et al., 2018).

In sum, this study addressed the question of what happens to microbes present in seeds during seedling germination and how are they distributed to above and below ground tissues. Their retention (and loss) and distribution patterns during seedling growth also provides some insight into why they are there. Because the productivity and health of agricultural systems depend greatly upon the functional processes carried out by the plant-associated microbiome, to further examine the question “what are they doing there?” will need further functional analysis of these core microbes. If their function is beneficial and given they are core, they may be persistent and represent valuable biologicals.

The findings of this research support the hypothesis that the process of germination changes the microbial community inherited from seeds and partitions it into the above and below ground tissues. Certain microbes remain associated with specific tissue compartment and accumulate there to build a core microbiome. Most importantly, the effect of rice genotype, growth location and harvest year are not as strong a driving force as tissue compartment on shaping the microbial community. The common core microbiome of rice seedlings revealed by this study offer promise that we can develop and apply universal microbial inoculant to benefit global rice production.

## Data Availability Statement

The datasets presented in this study can be found in online repositories. The names of the repository/repositories and accession number(s) can be found at: https://www.ncbi.nlm.nih.gov/, SAMN14836377–SAMN14836424.

## Author Contributions

MW and YO carried out the plant samples preparation, gDNA extraction, and amplicon library preparation. MW, AE, and MT analyzed the sequencing data. MW wrote the manuscript draft under the review and supervision of MT and RD. All authors conceived and planned the research.

## Conflict of Interest

The authors declare that the research was conducted in the absence of any commercial or financial relationships that could be construed as a potential conflict of interest.

## Footnotes

1 http://support.illumina.com/downloads/16s_metagenomic_sequencing_library_preparation.html

2 https://www.bioinformatics.babraham.ac.uk/projects/fastqc/

3 https://zenodo.org/record/1172783#.XvQNAmpKjfA

4 https://unite.ut.ee/repository.php

5 https://www.r-project.org/; https://rstudio.com/

6 https://digitalinsights.qiagen.com

7 https://www.graphpad.com/scientific-software/prism/
