# Supplementary material for: Dynamic Changes in the Microbiome of Rice During Shoot and Root Growth Derived From Seeds
Source: Front Microbiol. 2020 Sep 8;11:559728. doi: 10.3389/fmicb.2020.559728 (PMC7506108; doi:10.3389/fmicb.2020.559728)
Supplement: Supplementary file 1 [file Table_1.docx]

Supplementary Material

# Supplementary Table 1

Number of raw and processed reads of bacterial and fungal data from each rice seedling sample.

# Supplementary Table 2

Distribution of reads among seedling compartments. Each compartment contains reads from six samples.

|  | | | | | | |
| --- | --- | --- | --- | --- | --- | --- |
| **Compartment** | **Raw** | **Quality Control**  **Bacteria Fungi** | | |  | |
| Shoot_Surface | 5348854 | 1176571 | 1004412 |  | |  |
| Shoot_Endosphere | 5187683 | 1349834 | 2414191 |  |  |  |
| Root_Surface | 3041230 | 619908 | 1966 |  |  |  |
| Root_Endosphere | 4730964 | 955602 | 2496917 |  |  |  |
| **TOTAL** | **18308731** | **4101915** | **5917486** |  | |  |

#

# Supplementary Table 3

Alpha diversity of bacterial ASVs within samples pooled based on rice genotype, growth location and harvest year.


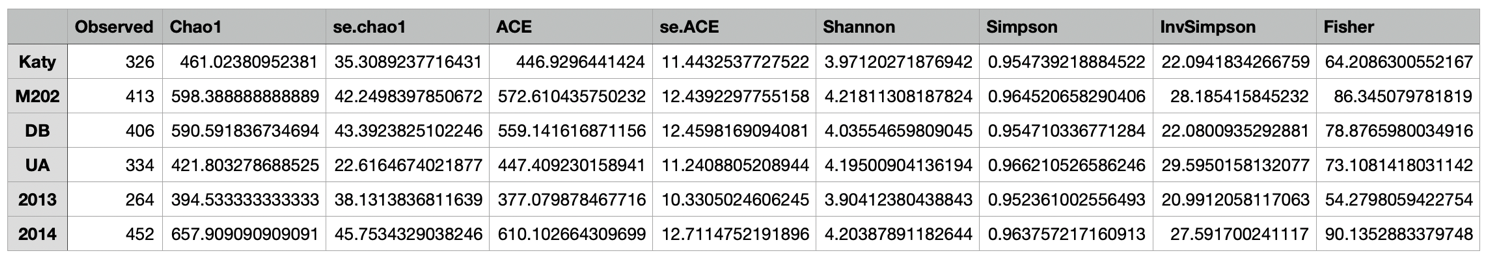


# Supplementary Table 4

Alpha diversity of fungal ASVs within samples pooled based on rice genotype, growth location and harvest year.


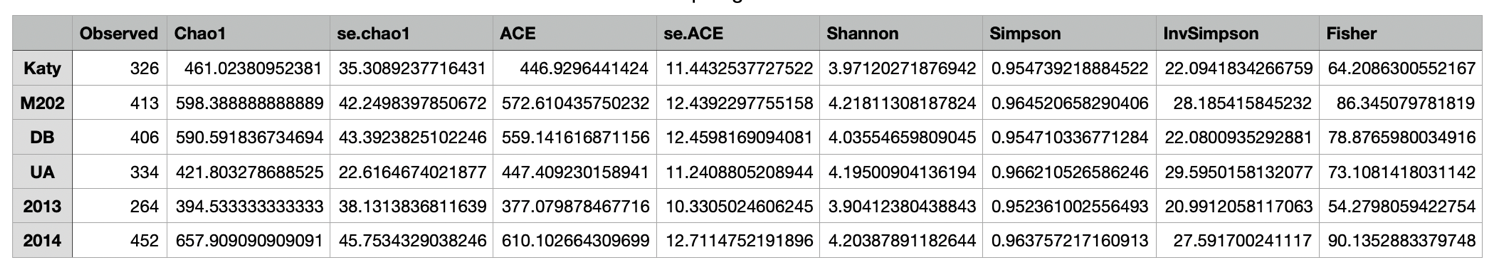


# Supplementary Table 5

Top bacterial taxa with >0.1% total reads. All bacterial ASVs with greater than 0.1% of the total reads sorted by their k-means clustering assignment. The headers represent the sample factors. The values are the average relative abundance for the samples pooled based on the respective factor. Tissue compartment: G: Grain, H: Husk; OG: Outer Grain; OH: Outer Husk; RE: Root Endosphere, RS: Root Surface; SE: Shoot Endosphere; SS: Shoot Surface.

#

# Supplementary Table 6

Top fungal taxa with >0.1% total reads. All fungal taxa with greater than 0.1% of the total reads sorted by their k-means clustering assignment. The headers represent the sample factors. The values are the average relative abundance for the samples pooled based on the respective factor. Tissue compartment: G: Grain, H: Husk; OG: Outer Grain; OH: Outer Husk; RE: Root Endosphere, RS: Root Surface; SE: Shoot Endosphere; SS: Shoot Surface.


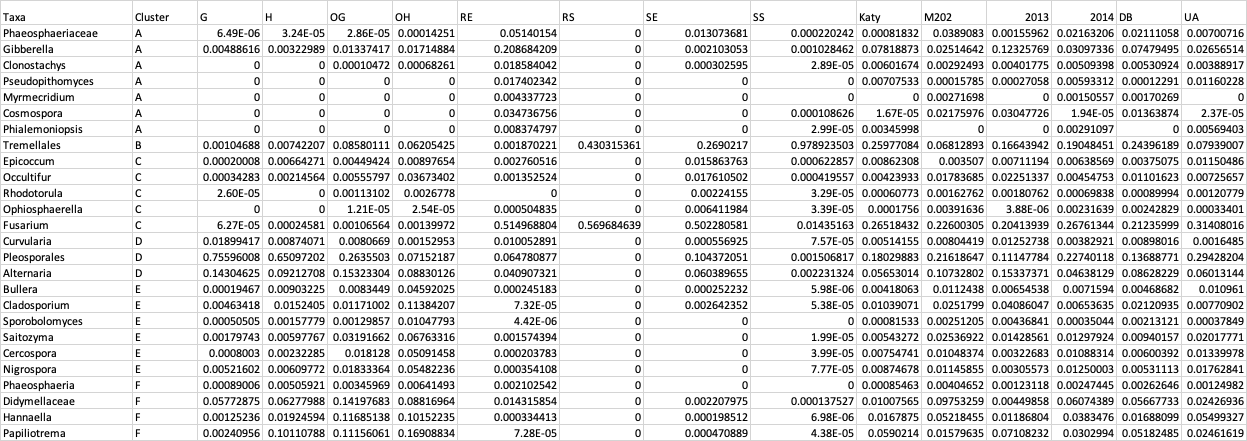


## Supplementary Figure 1

Venn diagram. Distribution of unique bacterial ASVs grouped by different tissue compartments.


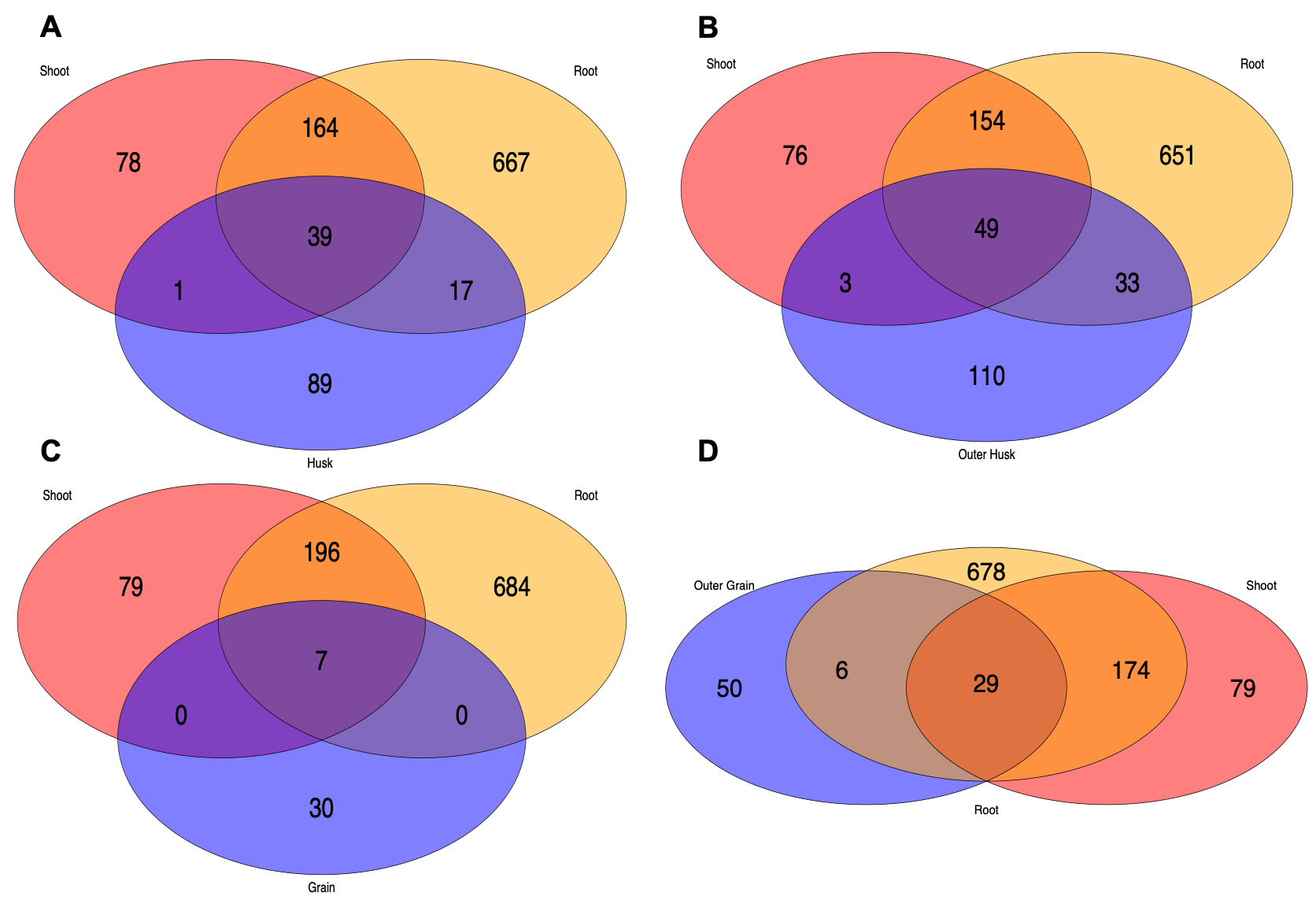


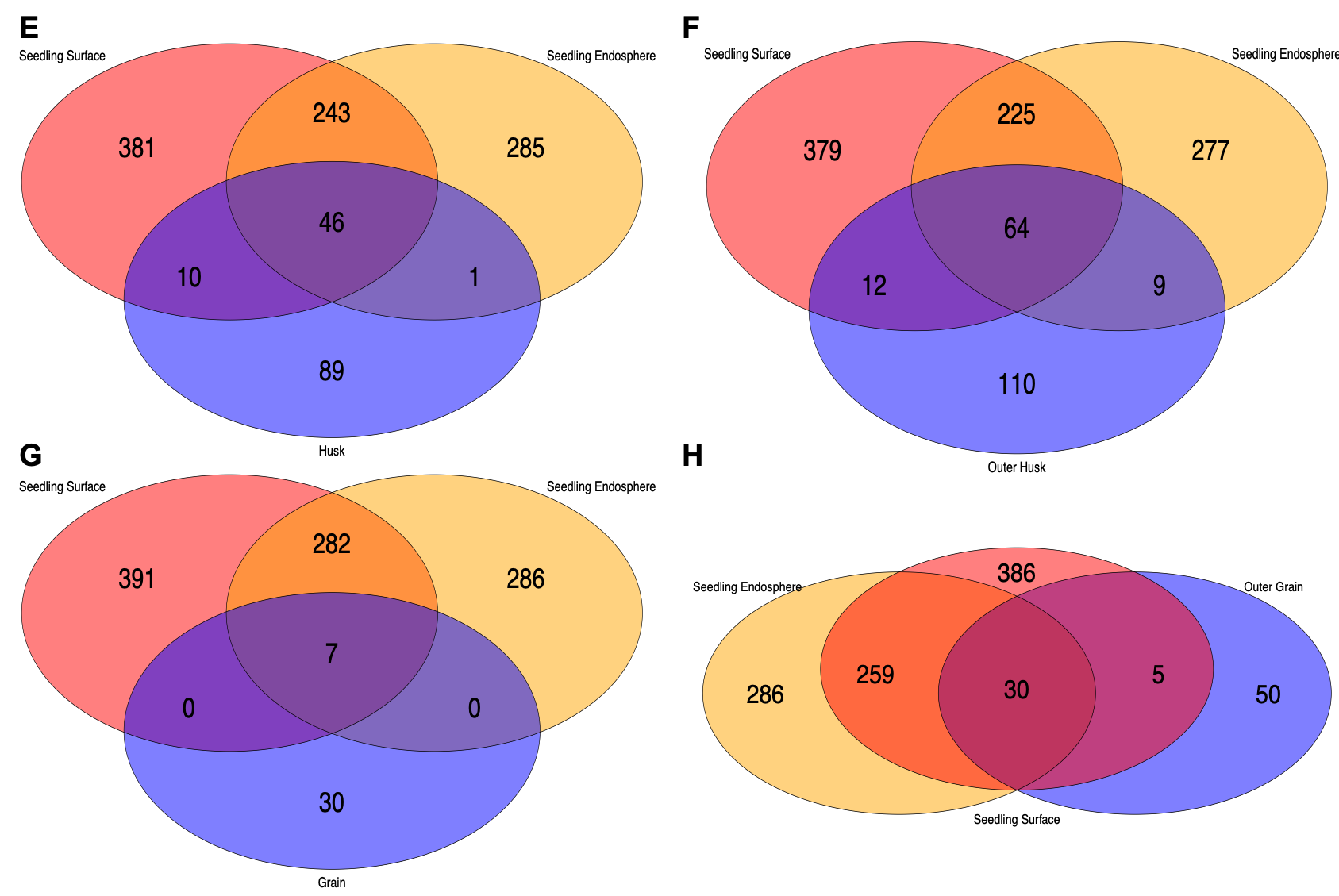


## Supplementary Figure 2

Venn diagram. Distribution of unique fungal ASVs grouped by different tissue compartments.


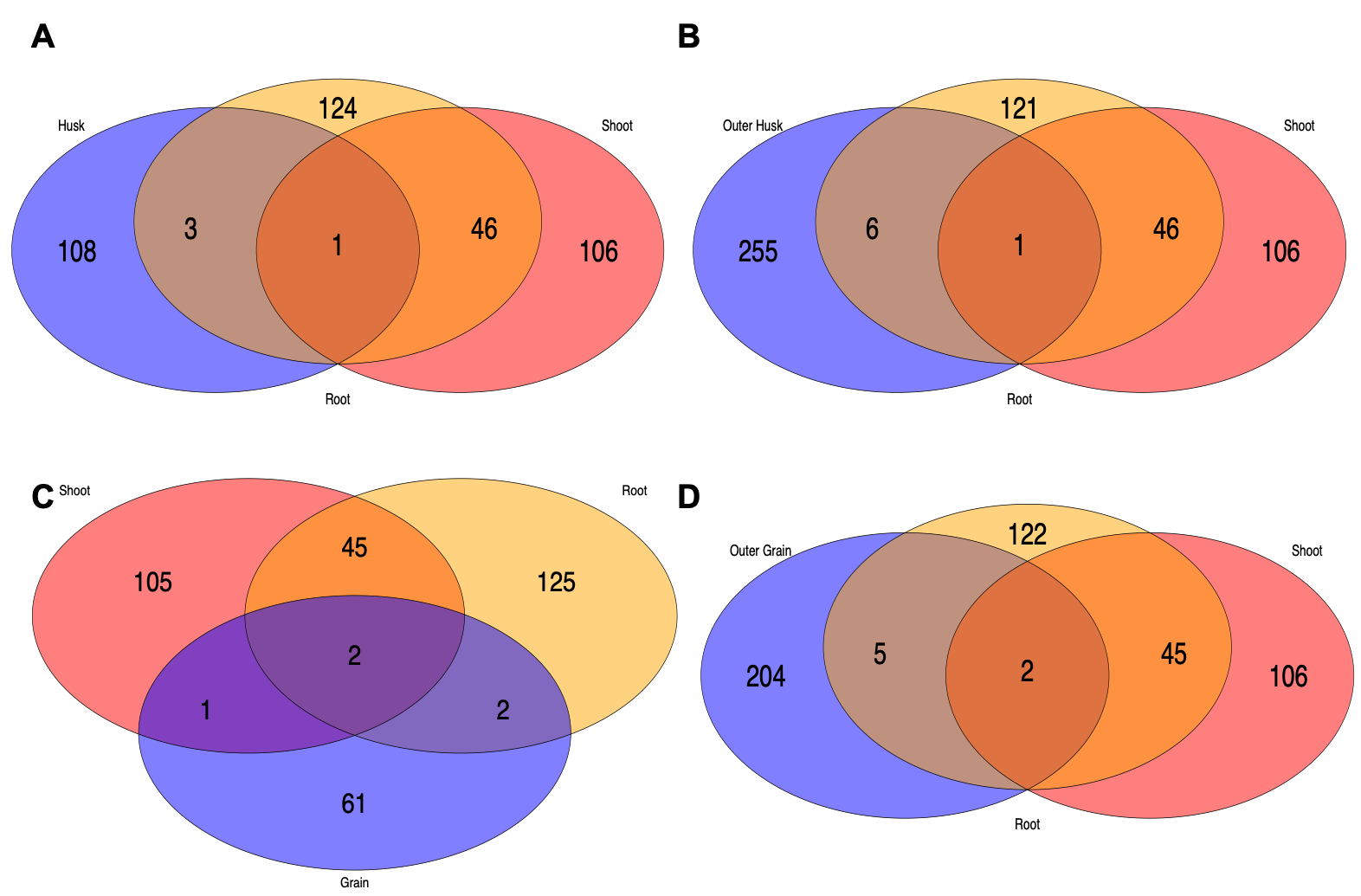


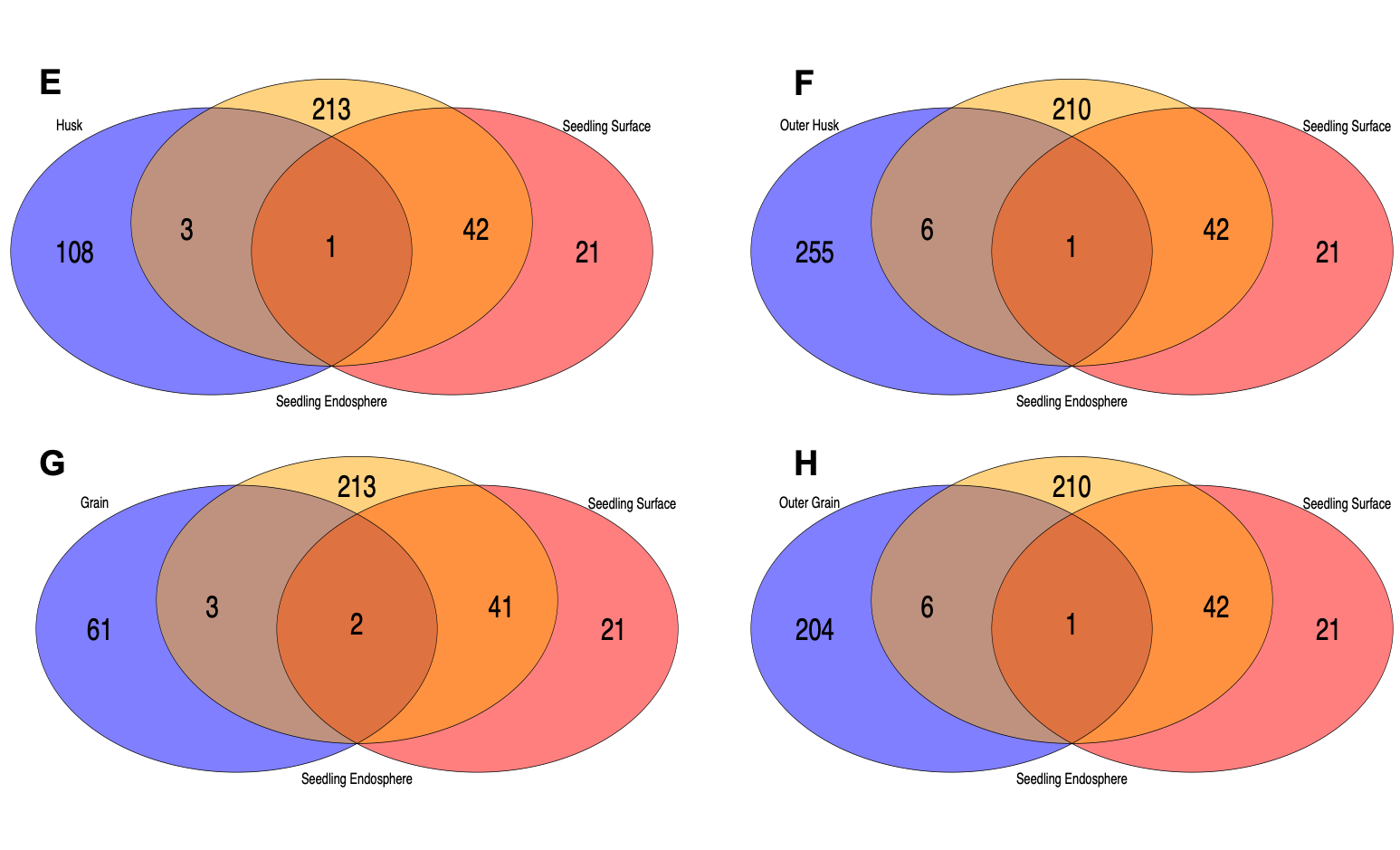


## Supplementary Figure 3

## Sunburst figure showing taxa proportion for Bacterial data.

##


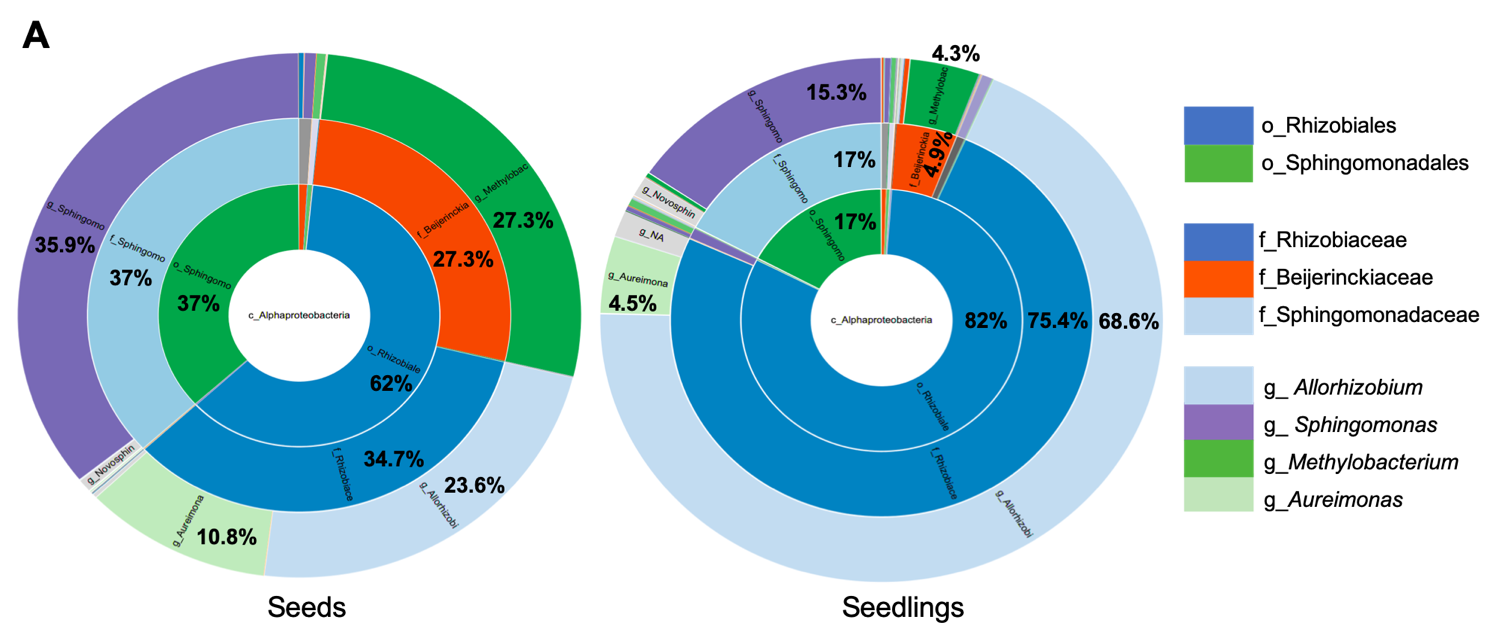


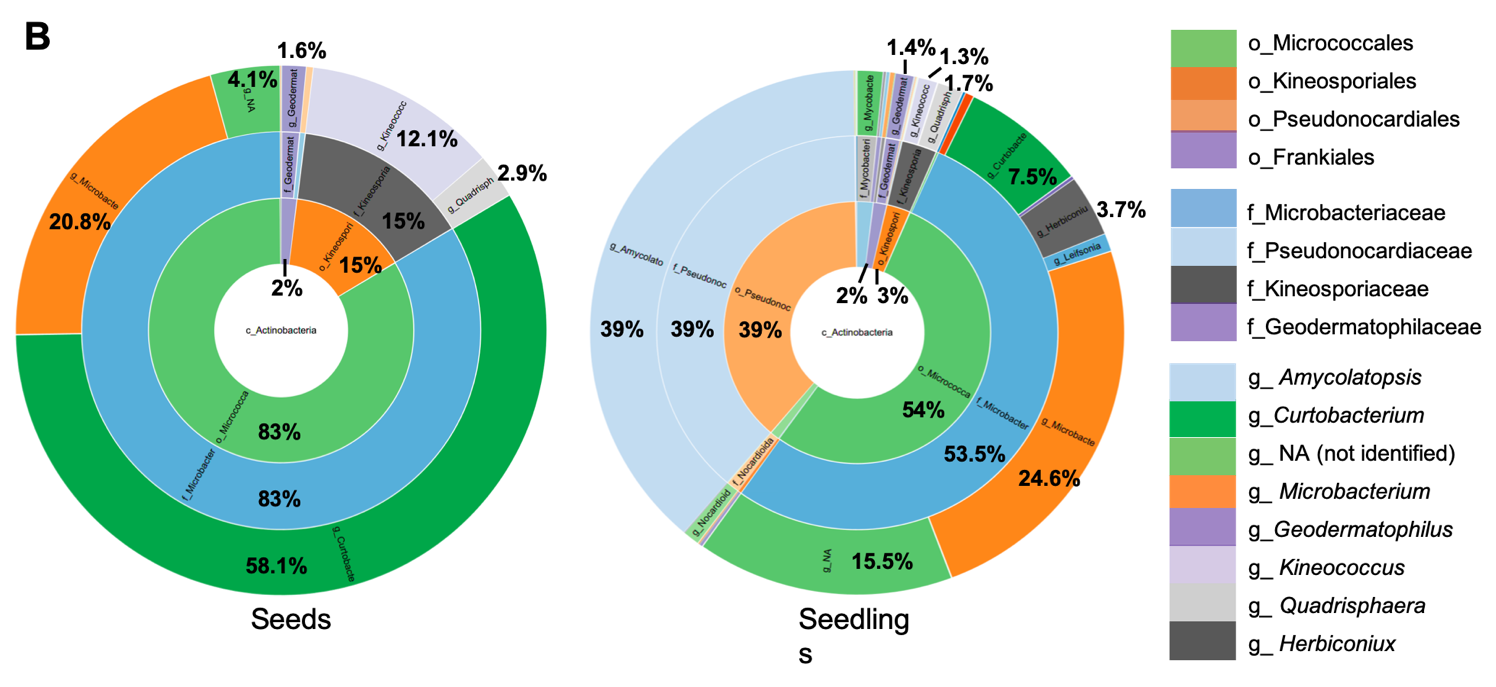


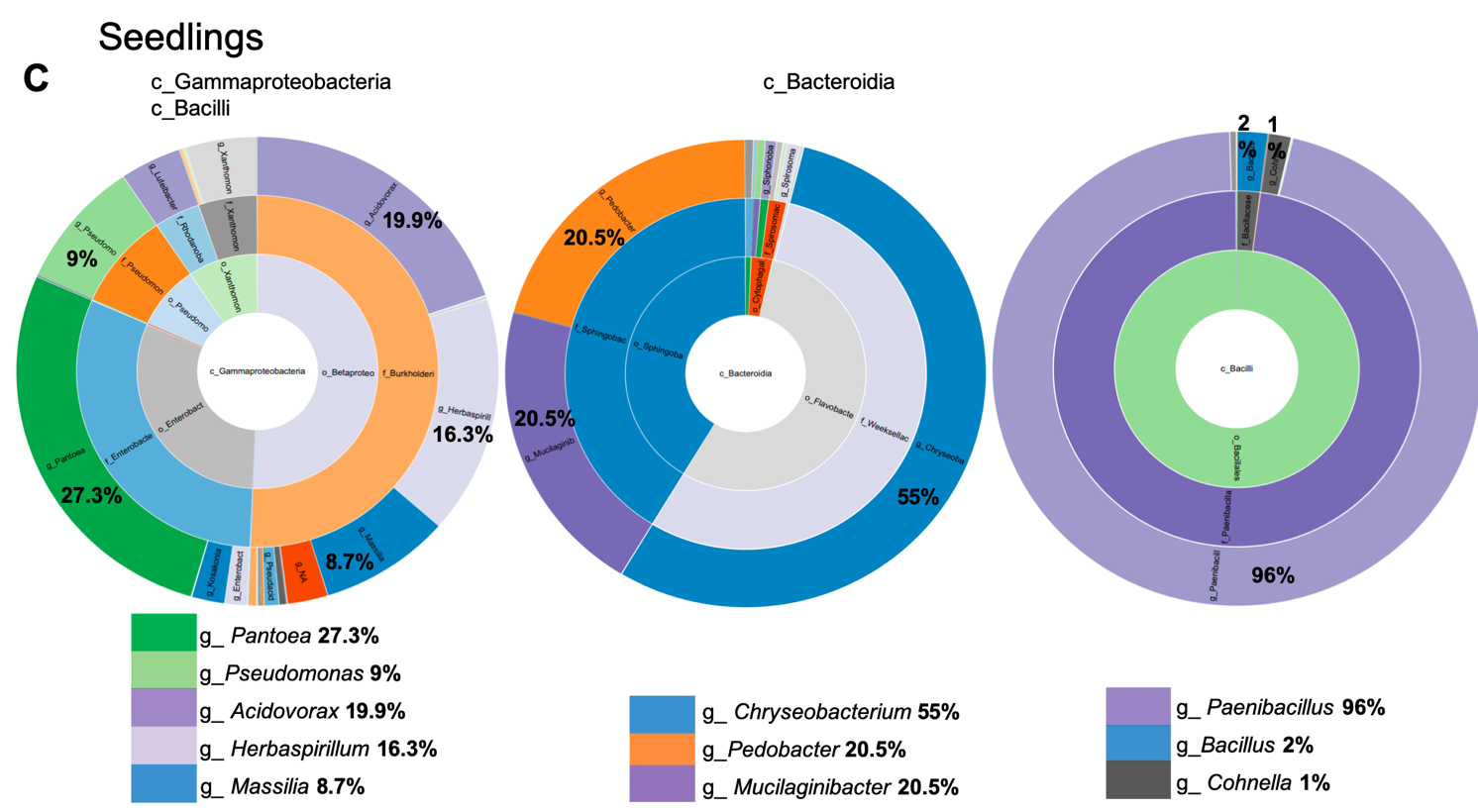


## Supplementary Figure 4

## Sunburst figure showing taxa proportion for fungal data.

##
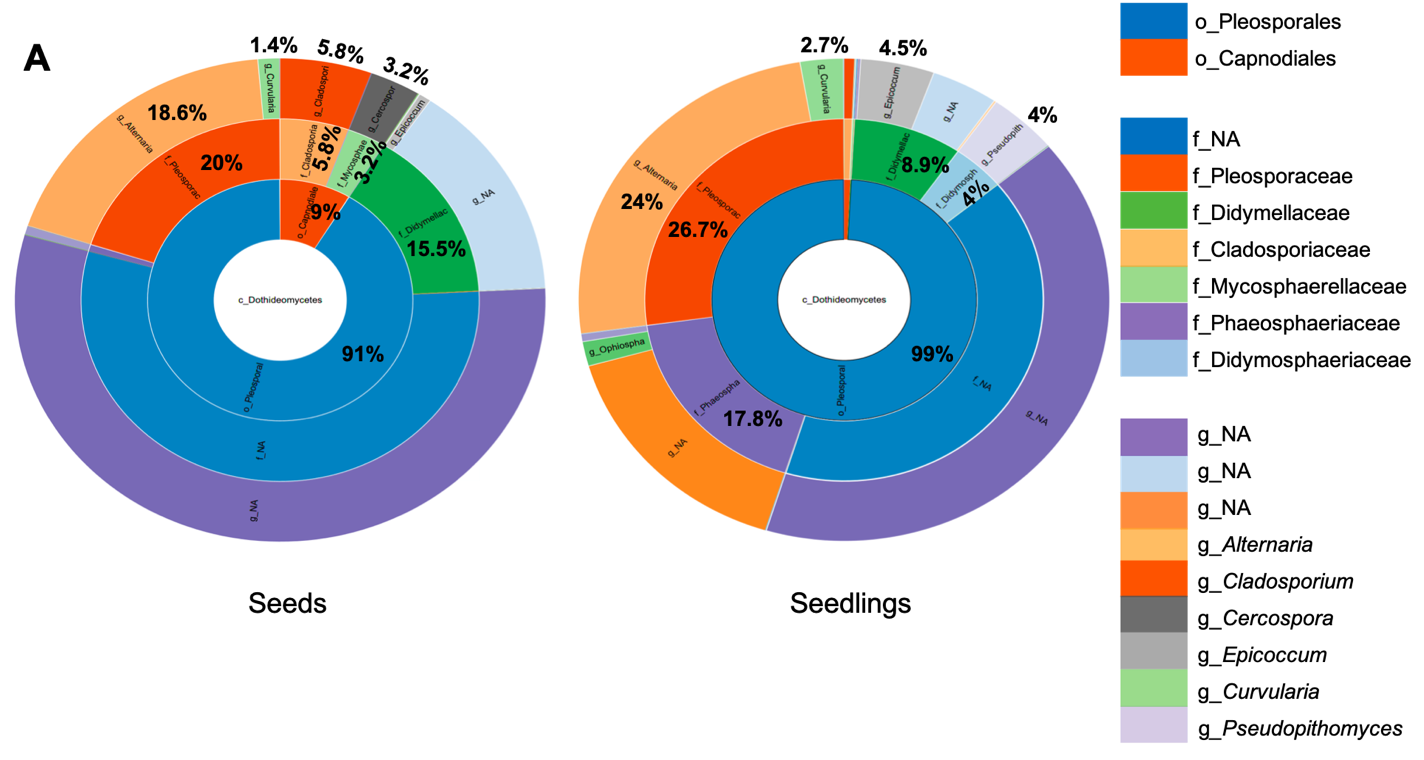


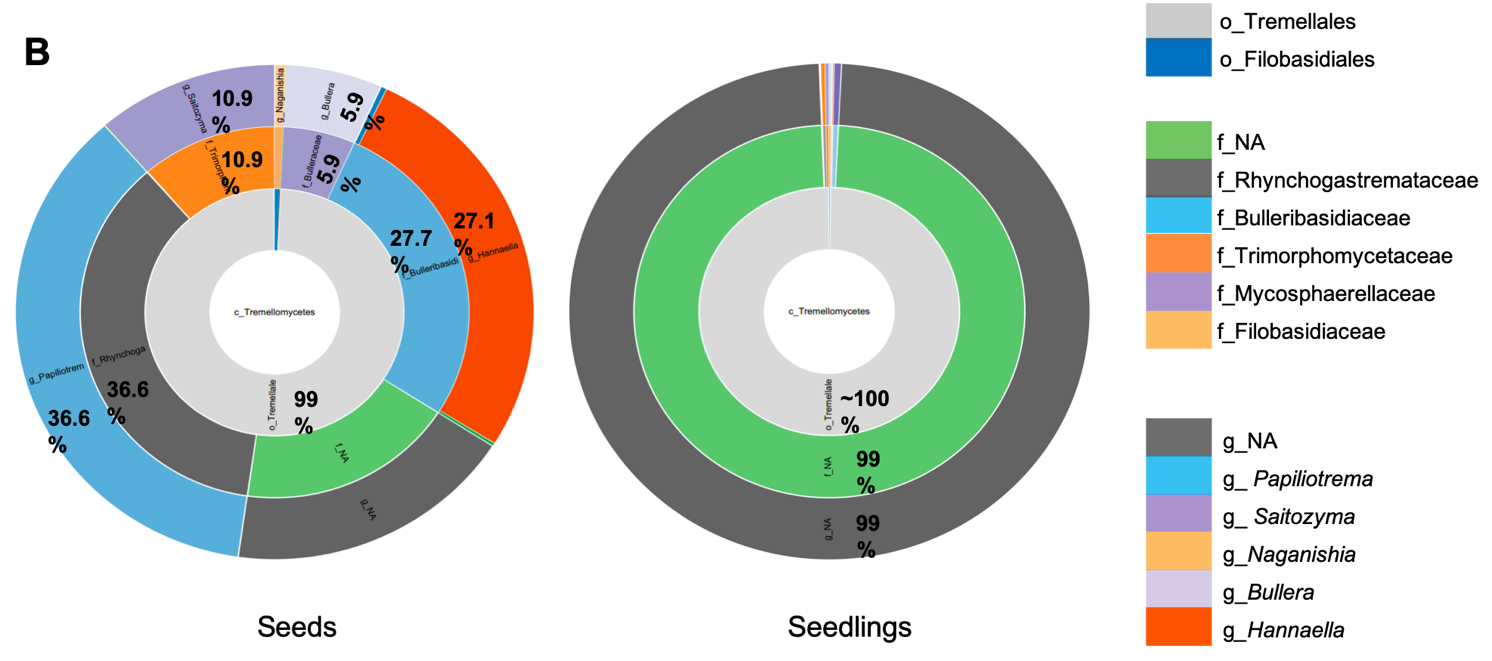


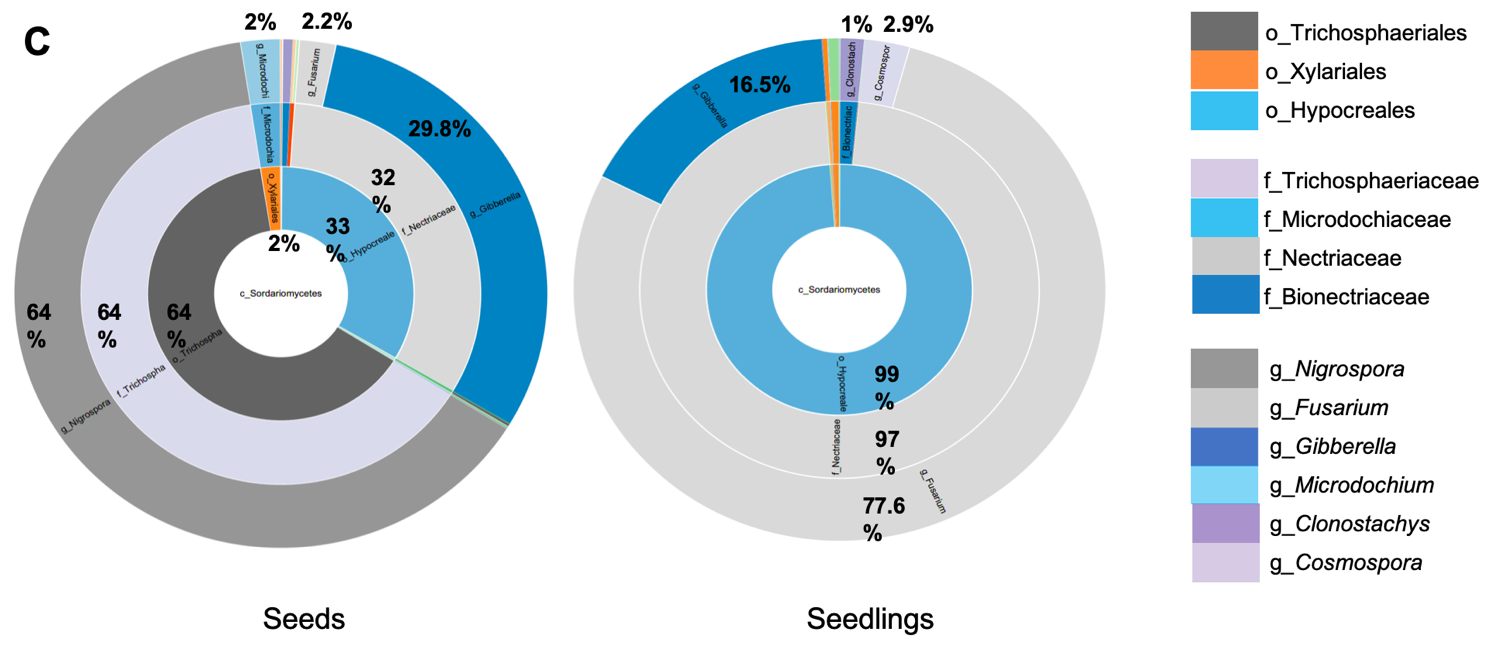


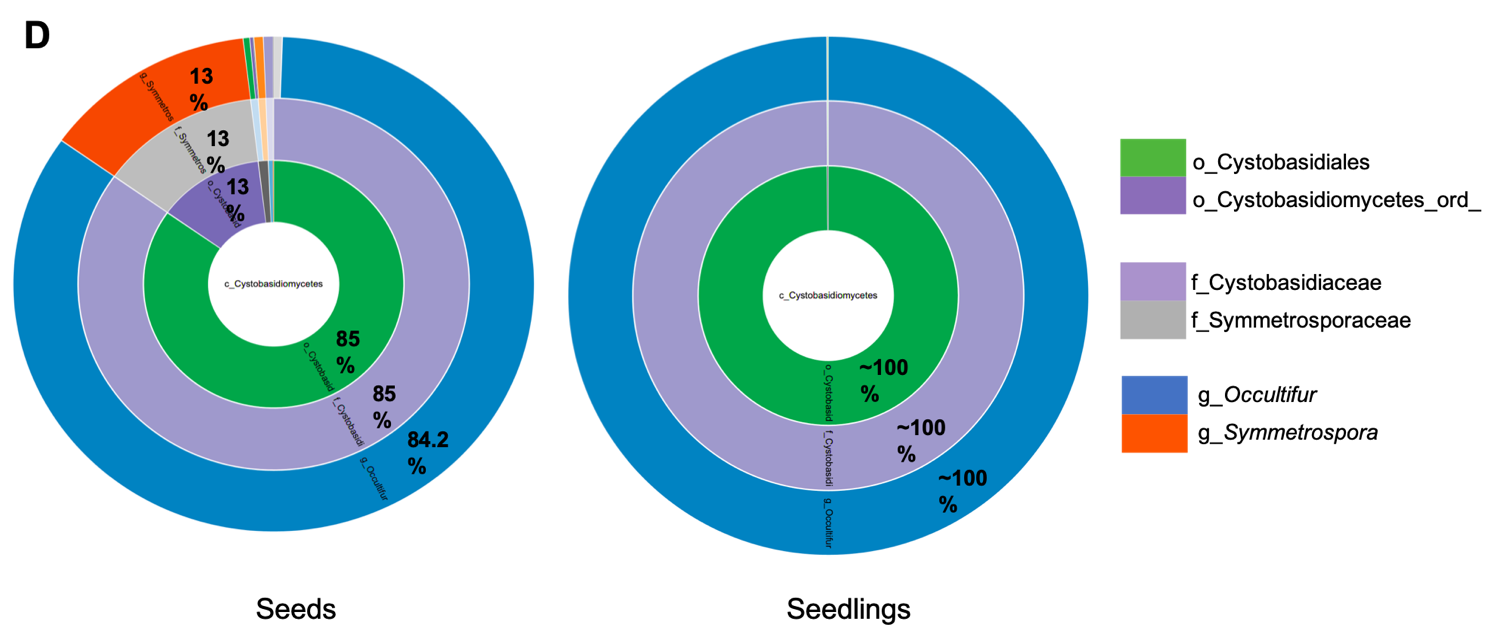


## Supplementary Figure 5

Bacterial PCoA for seedlings. Principal coordinate analysis (PCoA) performed on the unrarefied (A-D) and rarefied (E-F) seedling bacterial data, then colorized and shaped by different groups: tissue compartments (A, E); harvest year (B, F); location (C, G) and rice genotype (D, H).

## Supplementary Figure 6

Fungal PCoA for seedlings. Principal coordinate analysis (PCoA) performed on the unrarefied (A-D) and rarefied (E-F) seedling fungal data, then colorized and shaped by different groups: tissue compartments (A, E); harvest year (B, F); location (C, G) and rice genotype (D, H).

## Supplementary Figure 7

Cluster Dendrogram for bacterial (A) and fungal (B) data. Numbers represent different taxa while red lines separate 6 different clusters.

**A**

A B C D E F

**B**

A B C D E F

**
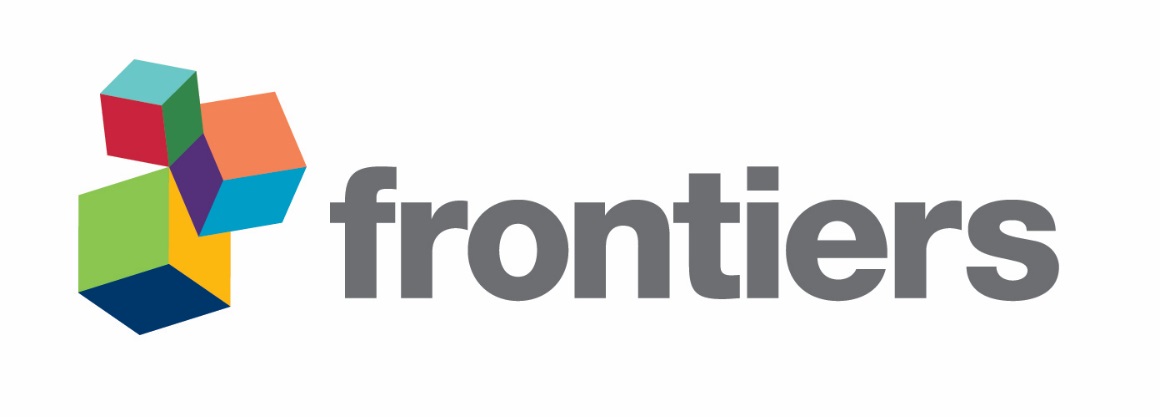
**
